# GST activity and membrane lipid saturation prevents mesotrione-induced cellular damage in *Pantoea ananatis*

**DOI:** 10.1186/s13568-016-0240-x

**Published:** 2016-09-13

**Authors:** Lilian P. Prione, Luiz R. Olchanheski, Leandro D. Tullio, Bruno C. E. Santo, Péricles M. Reche, Paula F. Martins, Giselle Carvalho, Ivo M. Demiate, Sônia A. V. Pileggi, Manuella N. Dourado, Rosilene A. Prestes, Michael J. Sadowsky, Ricardo A. Azevedo, Marcos Pileggi

**Affiliations:** 1 Departamento de Biologia Estrutural, Molecular e Genética, Universidade Estadual de Ponta Grossa, Campus Universitário de Uvaranas, Av. Carlos Cavalcanti, 4748, Ponta Grossa, Paraná 84030-900 Brazil; 2 Departamento de Microbiologia, Instituto de Ciências Biomédicas, Universidade de São Paulo, São Paulo, São Paulo 05508-000 Brazil; 3 Departamento de Enfermagem e Saúde Pública, Universidade Estadual de Ponta Grossa, Campus Universitário de Uvaranas, Av. Carlos Cavalcanti, 4748, Ponta Grossa, Paraná 84030-900 Brazil; 4 Departamento de Genética, Escola Superior de Agricultura Luiz de Queiroz, Universidade de São Paulo, Piracicaba, São Paulo Brazil; 5 Departamento de Engenharia de Alimentos, Universidade Estadual de Ponta Grossa, UEPG, Av. Carlos Cavalcanti, 4748, Ponta Grossa, PR 84030-900 Brazil; 6 Universidade Tecnológica Federal do Paraná, UTFPR, Campus Ponta Grossa, Av. Monteiro Lobato, Ponta Grossa, PR 84016-210 Brazil; 7 Department of Soil, Water, and Climate, and The BioTechnology Institute, University of Minnesota, Saint Paul, MN 55108 USA

**Keywords:** Herbicide degradation, Lipid peroxidation, Mesotrione, Fatty acid saturation, Glutathione-S-transferase

## Abstract

Callisto^®^, containing the active ingredient mesotrione (2-[4-methylsulfonyl-2-nitrobenzoyl]1,3-cyclohenanedione), is a selective herbicide that controls weeds in corn crops and is a potential environmental contaminant. The objective of this work was to evaluate enzymatic and structural changes in *Pantoea ananatis,* a strain isolated from water, in response to exposure to this herbicide. Despite degradation of mesotrione, probably due a glutathione-S-transferase (GST) pathway in *Pantoea ananatis,* this herbicide induced oxidative stress by increasing hydrogen peroxide production. Thiol fragments, eventually produced after mesotrione degradation, could be involved in increased GST activity. Nevertheless, there was no peroxidation damage related to this production, as malondialdehyde (MDA) synthesis, which is due to lipid peroxidation, was highest in the controls, followed by the mesotrione- and Callisto^®^-treated cultures at log growth phase. Therefore, *P. ananatis* can tolerate and grow in the presence of the herbicide, probably due an efficient control of oxidative stress by a polymorphic catalase system. MDA rates depend on lipid saturation due to a pattern change to a higher level of saturation. These changes are likely related to the formation of GST-mesotrione conjugates and mesotrione degradation-specific metabolites and to the presence of cytotoxic adjuvants. These features may shift lipid membrane saturation, possibly providing a protective effect to bacteria through an increase in membrane impermeability. This response system in *P. ananatis* provides a novel model for bacterial herbicide tolerance and adaptation in the environment.

**Electronic supplementary material:**

The online version of this article (doi:10.1186/s13568-016-0240-x) contains supplementary material, which is available to authorized users.

## Introduction

Pesticides have been widely used to increase crop production, yet there are concerns about the adverse effects that these chemicals have on wildlife because many agrochemicals are not readily degraded by microorganisms (Copley 2009). It is estimated that ~140,000 tons of synthetic pesticides are applied annually in the USA, and approximately 300 different pesticides have been reported to be contaminants of European food products (Bjørling-Poulsen et al. 2008). The metabolism and genetic diversity of microbial communities, in both soil and plants, can be altered by pesticides as well as by the presence and expression of genes encoding enzymes that facilitate herbicide degradation (Simon and Daniel 2011; Tétard-Jones and Robert 2015). The biological degradation of pesticides appears to be one of the best strategies for potentially removing xenobiotics from the environment. Indeed, the application of herbicide biodegradation and bioremediation methodologies to pesticide-contaminated environments is one of the most promising areas of biotechnological research (Martins et al. 2007).

Mesotrione (2-[4-methylsulfonyl-2-nitrobenzoyl]1,3-cyclohenanedione), the active ingredient of the herbicide Callisto^®^, is used for selective pre- and post-emergent control of broadleaf weeds in corn crops (Batisson et al. 2009). This chemical, which is naturally produced by the plant *Callistemon citrinus*, functions by inhibiting the enzyme 4-hydroxyphenylpyruvate dioxygenase (HPPD) and thus interferes with carotenoids synthesis (Mitchell et al. 2001). Mesotrione has been shown to be an environmental contaminant (Stoob et al. 2005). Both 4-methylsulfonyl-2-nitrobenzoic acid (MNBA) and 2-amino-4-methylsulfonyl benzoic acid (AMBA) have been described as products of mesotrione degradation by *Bacillus* sp., (Durand et al. 2006) with AMBA being more cytotoxic than the active ingredient mesotrione (Mitchell et al. 2001; Bonnet et al. 2008). Furthermore, a recent study reported that other products, in addition to AMBA, are produced through mesotrione degradation by *Pantoea ananatis* (Pileggi et al. 2012).

Oxidative stress is characterized by an increase in the production of reactive oxygen species (ROS) to a level greater than the cell’s ability to defend against them (Ghelfi et al. 2011; Peters et al. 2014). Most ROS in bacteria are derived from the sequential reduction of O_2_ catalyzed by enzymes in the electron transport chain associated with the plasma membrane (Lushchak 2001). Highly reactive products of aerobic metabolism, such as hydrogen peroxide (H_2_O_2_), superoxide (O_2_^•^) and hydroxyl (OH^•^) radicals, can damage DNA, RNA, proteins and lipids (Gratão et al. 2005), and antioxidant systems, such as the enzymes catalase, peroxidase and glutathione reductase (Olchanheski et al. 2014; Peters et al. 2014), are invoked to combat reactive oxygen intermediates. For example, bacteria exposed to the herbicide acetochlor at 62 and 620 mM concentrations exhibited an increase in lipid peroxidation by 39 and 34 %, respectively, suggesting that microorganisms can tolerate some cytotoxic agrochemicals via induction of antioxidant stress responses (Martins et al. 2011).

*Pantoea ananatis* CCT 7673 is a mesotrione-degrading bacterium that we previously isolated from water, together with 359 other mesotrione-tolerant microorganisms (Pileggi et al. 2012). Bacterial growth studies showed that *P. ananatis* tolerates high concentrations of mesotrione and Callisto^®^. high-performance liquid chromatography (HPLC) analysis demonstrated that *P. ananatis* CCT 7673 can degrade this herbicide within ~17 h, resulting in products that are less toxic than those produced by a *Bacillus* sp. strain (Crouzet et al. 2010). Despite the ability of *P. ananatis* to degrade mesotrione, this bacterium did not utilize the herbicide as a nutrient source for growth (Pileggi et al. 2012).

The aim of this current study was to determine the mechanism(s) by which *P. ananatis* resists the toxic effects of mesotrione and its commercial formulation Callisto^®^. We also examined whether mesotrione creates stress responses in *P. ananatis*, ultimately affecting cellular metabolism through enzymatic responses.

## Materials and methods

### Chemicals

A commercial formulation of Callisto^®^, containing 48 % mesotrione (the active ingredient) (Additional file 1) and adjuvants benzisotiazolin-1.2-3-one, 1-octanol, poly (oxy-1, 2-etanediil) and alpha-isodecyl-omega-hydroxy-phosphate, was used in this study (https://www.syngenta-crop.co.uk/products/callisto/summary.aspx). Mesotrione was kindly provided by Syngenta Crop Protection, Greensboro, NC (USA).

### Mesotrione-degrading strain

The bacterium used in this study, *P. ananatis* CCT 7673, was previously isolated at Capão da Onça School Farm-Ponta Grossa State University, Ponta Grossa-PR, Brazil, and was previously shown to be a mesotrione-degrading bacterium (Pileggi et al. 2012).

### Bacterial culture

The *P. ananatis* CCT 7673 was cultured in 900 mL Luria Broth (LB, Himedia, Mumbai, India) for 24 h at 30 °C. The cells were centrifuged, washed twice in phosphate-buffered saline, pH 7.0 (PBS: 8 g L^−1^ NaCl, 0.2 g L^−1^ KCl, 1.44 g L^−1^ Na_2_HPO_4_ and 0.24 g L^−1^ KH_2_PO_4_), and divided into nine separate flasks containing 50 mL of mineral medium (MM). The MM was composed of 10 mM potassium phosphate buffer, pH 7.0, supplemented with the following compounds (in g L^−1^): 3 NaNO_3_, 0.5 MgSO_4_, 0.5 KCl, 0.01 FeSO_4_, 0.04 CaCl_2_, 0.001 MnSO_4_, 0.4 glucose and 15 agar. Experiments were performed in triplicate under the following conditions: MM (control), Mesotrione Mineral Medium [MMM: MM plus 0.04 mM mesotrione, 1× Field Rate (FR), or the equivalent concentration used in agriculture, following the manufactory instructions], and Callisto Mineral Medium (CMM: MM plus 0.04 mM mesotrione in Callisto^®^). All cultures were incubated at 30 °C. The treatments were used in subsequent assays.

### Herbicide treatment and growth curve determination

Bacterial growth (600 µL) was measured spectrophotometrically at 600 nm using 600 µL of sample collected every 2 for 24 h. When the samples reached an OD of greater than 0.6, bacterial growth was also measured by dilution-plating on MM, MMM and CMM media.

### Cell viability

Bacterial suspensions were diluted in 0.9 % NaCl to 10^−7^ after 30 min and to 10^−8^ at 12, 17.5 and 19 h. Samples (100 µL) were spread-plated, in triplicate, onto Luria Agar (LA, Himedia, Mumbai, India) plates, and incubated at 30 °C for 24 h.

### Hydrogen peroxide quantification (H_2_O_2_)

Cells (100 mg) were homogenized in liquid nitrogen containing 0.1 % tricloroacetic acid (TCA) using a mortar and pestle and centrifuged at 11,600×*g* for 15 min at 4 °C. A 0.2 mL aliquot of the supernatant was added to 0.2 mL 100 mM phosphate buffer, pH 7.5; 0.8 mL of 1 M KI solution was added. The reaction was mixed and incubated in the dark at 4 °C for 1 h, followed by 20 min at room temperature. The absorbance was measured at 390 nm, and the results are expressed as mmol H_2_O_2_/10^6^ C.F.U (Dourado et al. 2013).

### Lipid peroxidation

Lipid peroxidation was quantified by estimating the levels of thiobarbituric acid (TBA)-reactive substances, as previously described (Heath and Packer 1968). The concentration of malondialdehyde (MDA) was monitored at 535 and 600 nm using a Perkin Elmer Lambda 40 spectrophotometer, and the MDA concentration was calculated using a molar extinction coefficient of 155 mM cm^−1^.

### Membrane lipid evaluation

After 12, 17.5 and 19 h of incubation, bacterial samples were centrifuged for 5 min at 11,600×*g* at 4 °C, and the precipitate was frozen. The material was lyophilized for 15 h; a solution of chloroform, methanol and water (1: 2: 0.8) was added, and the mixture was stirred at 180 rpm for 18 h at room temperature. After this period, a mixture of chloroform, methanol and water (1: 1: 0.9) was added to a final volume of 5.8 mL (Bligh et al. 1959). The chloroform phase was collected, and the membrane lipid fraction was analyzed by Fourier Transformed Infrared Spectroscopy (FTIR), from 400 to 4000 cm^−1^. Statistical analysis was performed using principal component analysis (PCA) and partial least squares (PLS), as implemented in the Pirouette v 4.0 software (Infometrix, Bothell, WA, EUA). PCA was performed using the average value of triplicates of the spectra, and PLS was performed with the value of each triplicate.

### Protein extraction and quantification

Total proteins were extracted, in triplicate, from *P. ananatis* CCT 7673 cells after 12 and 24 h of incubation after each treatment. Antioxidant enzymes were isolated as follows, at 4 °C, unless otherwise stated. The culture was centrifuged at 10,000×*g* for 10 min. The pellet was ground in liquid nitrogen using a mortar and pestle and homogenized (10:1, v/w) in 100 mM potassium phosphate buffer (pH 7.5) containing 1 mM ethylenediaminetetraacetic acid (EDTA), 3 mM DL-dithiothreitol and 5 % (w/v) polyvinylpolypyrrolidone. The homogenate was centrifuged at 10,000×*g* for 30 min, and the supernatant was stored in separate aliquots at –80 °C prior to enzymatic activity assays. The protein concentration was determined as described in Bradford (1976), using bovine serum albumin (BSA) as the standard.

### Polyacrylamide gel electrophoresis (PAGE)

Electrophoresis was carried out using 12 % polyacrylamide gels, with a 4 % stacking gel, as previously described (Monteiro et al. 2011). Sodium dodecyl sulfate (SDS) was omitted for non-denaturing gels. Electrophoresis was carried out at 4 °C at a constant current of 15 mA gel^−1^ for 3 h for gels stained for superoxide dismutase (SOD) activity or for 21 h for gels stained for catalase (CAT) activity. Equal amounts of protein (20 µg) were loaded per lane onto non-denaturing PAGE gels. For SDS-PAGE analysis, gels were stained as previously described (Azevedo et al. 1998).

### SOD isoforms

Classification of superoxide dismutase (EC 1.15.1.1) isoforms was performed by non-denaturing PAGE (12 % gels) with 300 μg of protein from bacterial extracts, as described by Gratão et al. (2015). Prior to staining, the gel was divided vertically into three parts: the first part was maintained at 4 °C in 100 mM potassium phosphate buffer, pH 7.8; the second was immersed in 100 mL of the same buffer but containing 2 mM KCN and 1 mM EDTA; and the third was immersed in 100 mL of buffer containing 5 mM H_2_O_2_ and 1.0 mM EDTA. All of the steps were performed in the dark. Isoforms were classified as Cu/Zn-SOD, Fe-SOD or Mn-SOD (Cabiscol et al. 2000).

### SOD activity staining

SOD activity was assayed using non-denaturing PAGE gels, essentially as described (Gratão et al. 2015). Following electrophoresis, gels were rinsed in distilled deionized water and incubated in the dark for 30 min in 50 mM potassium phosphate buffer, pH 7.8, containing 1 mM ethylenediamine tetraacetic acid (EDTA), 0.005 mM riboflavin, 0.1 mM nitroblue tetrazolium and 0.3 % N,N,N′,N′-tetramethylethylenediamine. One unit of bovine liver SOD (Sigma, St. Louis, MO, USA) was included as a positive control for the chemical reaction. The gels were immersed in water until the achromatic bands of SOD were revealed (Gratão et al. 2012).

### Catalase activity staining

Catalase (EC 1.11.1.6) activity was observed after protein separation by non-denaturing electrophoresis. Following staining, the gels were incubated in 0.003 % H_2_O_2_ for 10 min and developed in a 1 % (m/v) FeCl_3_ and 1 % K_3_Fe (CN_6_) solution for 10 min. One unit of bovine liver CAT (Sigma, St. Louis, USA) was used as a control for the chemical reaction (Boaretto et al. 2014).

### Glutathione-S-transferase (GST) assays

The activity of GST was assayed in a reaction mixture containing 0.1 M potassium phosphate buffer, pH 6.5, 0.1 mM glutathione sulfhydryl (GSH), and 0.04 M 1-cloro-2,4-dinitrobenzene (CDNB), as previously described (Ghelfi et al. 2011). The CDNB was mixed with buffer and GSH, and 25 µL of protein extract was added. The reaction was initiated 1 min after the mixture was prepared and incubated at 30 °C. Activity was determined by monitoring the kinetics of the formation of 1, 2-dichloro-4-nitrobenzene (DCNB) in samples for 3 min using a spectrophotometer at 610 nm. The results are expressed as µmol/min/mg protein.

### Statistical analysis

Data on H_2_O_2_, cell viability, MDA quantification and GST activity were obtained, in triplicate, for each treatment and analyzed statistically using a randomized complete design. The significance of differences was assessed by analysis of variance (p < 0.05). Statistical analyses were performed with Stata 12 software using factor analysis and the ANOVA test for comparisons between strains, treatments, and growth periods, and using the Bonferroni’s test for the analyses. We also used the Bartlett’s Test for inequality of population variances and Mann–Whitney/Wilcoxon Two-Sample Test (Kruskal–Wallis test for two groups).

## Results

**Fig. 1:**
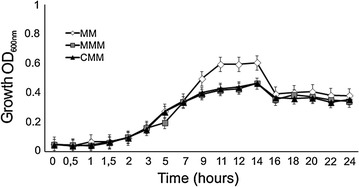
Growth (determined via OD_600 nm_) of *P. ananatis* CCT 7673 in control (MM), mesotrione-treated (MMM) and Callisto^®^-treated (CMM) cultures

Experimental pilot tests were conducted to determine the best incubation time of analysis. Two sets of time period were selected based on the growth curves (Fig. 1) and mesotrione degradation by *P. ananatis*. Based on associations with mesotrione degradation, quantification of H_2_O_2_, viability, GST, MDA and lipid saturation was performed at 12, 17.5 and 19 h (the late log phase, peak of mesotrione degradation and early stationary phases, respectively). In contrast, CAT and SOD activities were analyzed only at 12 and 24 h (the late log, before the mesotrione degradation peak, and stationary phases, after mesotrione degradation, respectively) because they were not related to the mesotrione degradation peak.

### Evaluation of peroxide levels

**Fig. 2:**
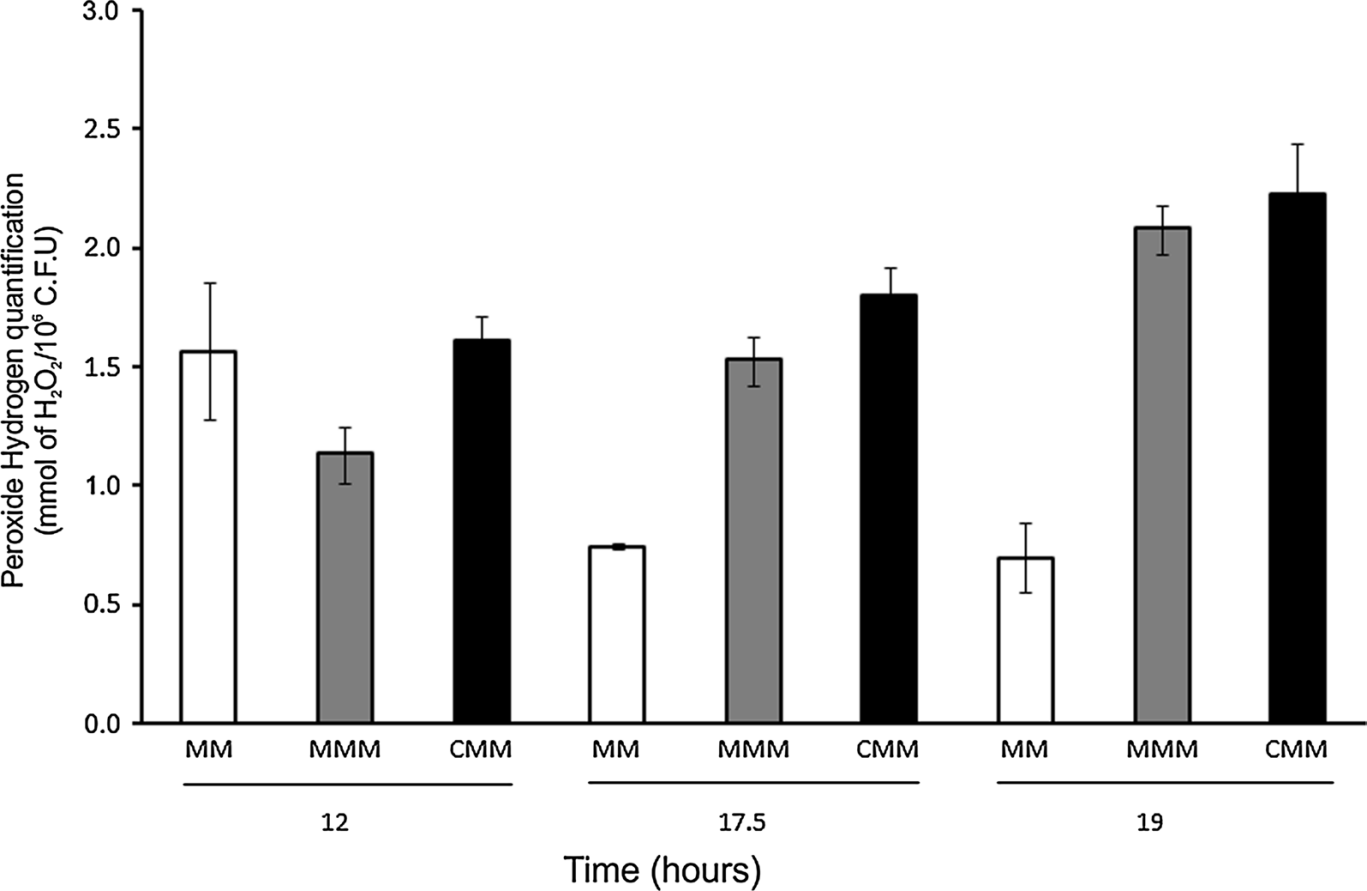
H_2_O_2_ quantification (representing oxidative stress) in control (MM)-, mesotrione (MMM)- and Callisto^®^ (CMM)-treated cultures after 12, 17.5 and 19 h. *Error bars* represent standard errors on means

The peroxide levels found in in *P. ananatis,* in response to mesotrione and Callisto^®^, are shown in Fig. 2.

### Viability and bacterial growth

**Fig. 3:**
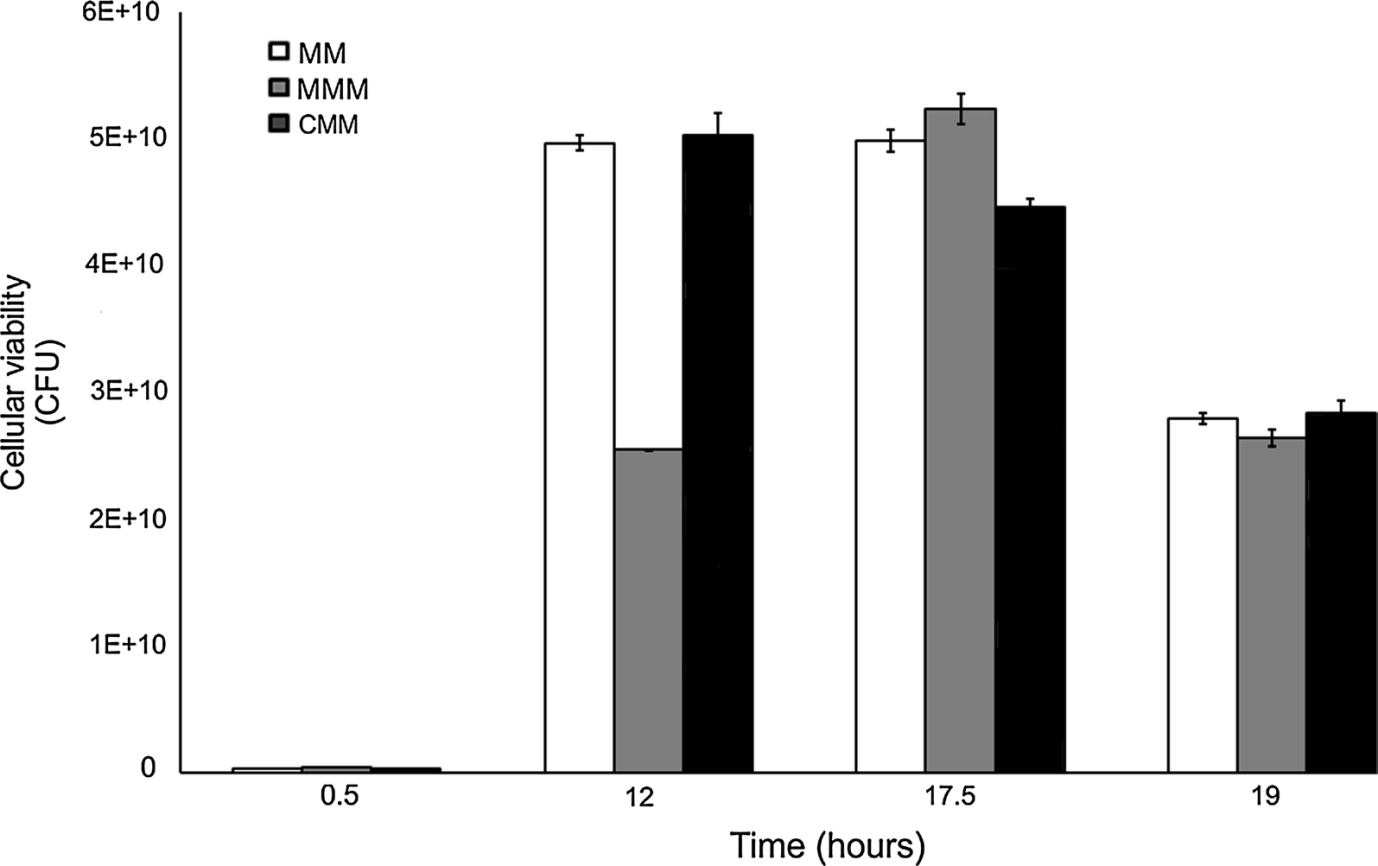
Viability data (CFU, colony forming unit) of *P. ananatis* CCT 7673 in control (MM), mesotrione-treated (MMM) and Callisto^®^-treated (CMM) cultures

The results of viability and bacterial growth experiments in MM (control) as well as MMM and CMM are shown in Figs. 1 and 3, respectively.

### SOD profile

**Fig. 4:**
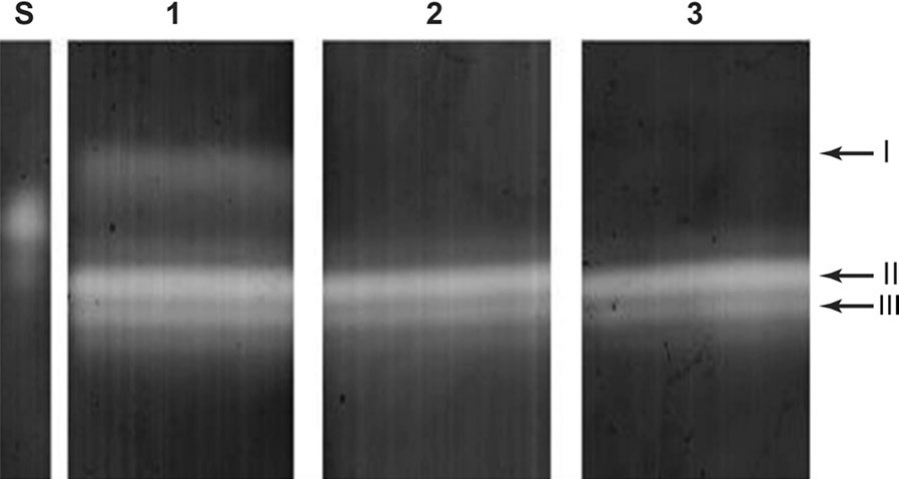
Classification of SOD isoforms following non-denaturing PAGE analysis. *Lane S* shows standard, *lane 1* corresponds to SOD activity in control, *lane 2* in the presence of 2 mM potassium cyanide, and *lane 3* with 5 mM H_2_O_2_. Band I corresponds to Cu/Zn-SOD. Bands II and III correspond to Mn-SOD

Classification of SOD isoforms was performed by non-denaturing PAGE. The Cu/Zn-SOD isoform is indicated by band I, and Mn-SOD by bands II and III (Fig. 4).

**Fig. 5:**
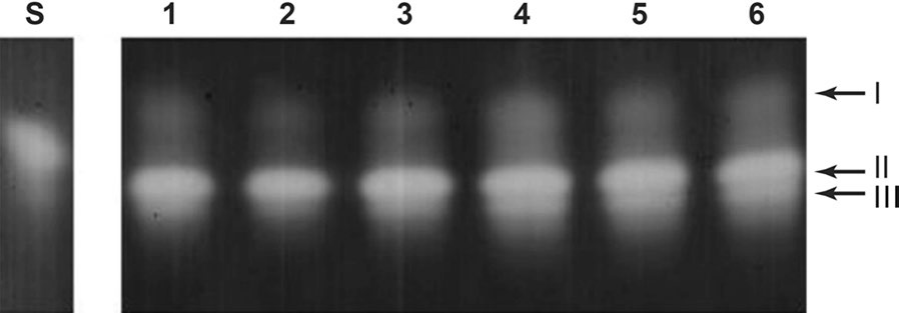
SOD activity staining following non-denaturing PAGE analysis of *P. ananatis* CCT 7673 extracts. *Lane S* shows SOD standard, *lanes 1* and *4* correspond to control, *lanes 2* and *5* to mesotrione treatment, and* lanes* 3 and 6 to Callisto^®^ treatment after 12 (*lanes 1*, *2* and *3*) and 24 h (*lanes 4*, *5* and *6*). Band I corresponds to Cu/Zn-SOD. Bands II and III correspond to Mn-SOD

Three SOD bands were found to be active in *P. ananatis* CCT 7673 exposed to the herbicides (Fig. 5), and all bands were more clearly present after 24 h of treatment.

### CAT activity and gel profiles

**Fig. 6:**
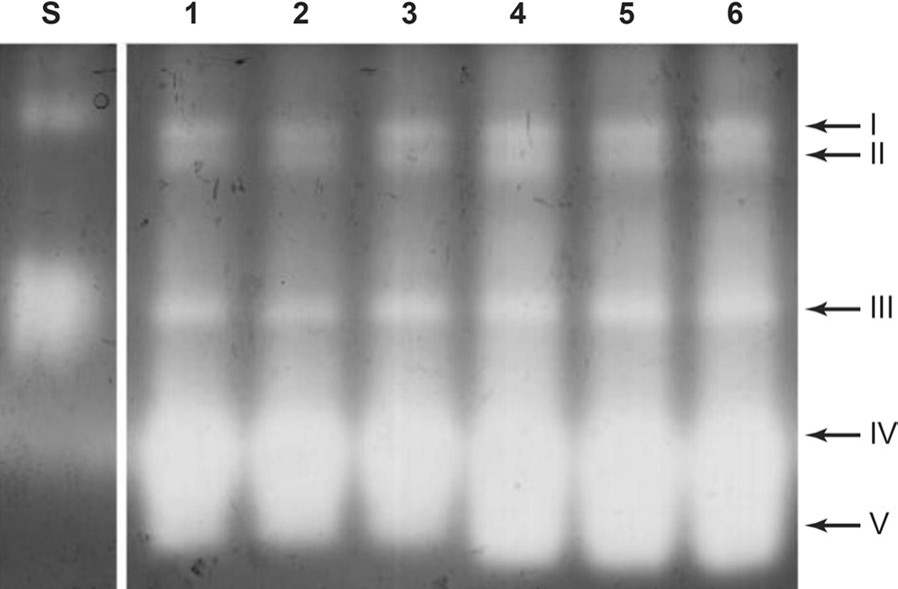
CAT activity staining following non-denaturing PAGE analysis of *P. ananatis* CCT 7673 extracts. *Lane S* shows SOD standard, *lanes 1* and *4* correspond to control, *lanes 2* and *5* to mesotrione treatment, and *lanes 3* and *6* to Callisto^®^ treatment after 12 (*lanes 1*, *2* and *3*) and 24 h (*lanes 4*, *5* and *6*)

The CAT activity in *P. ananatis* CCT 7673 at 12 and 24 h in mesotrione and Callisto^®^ treatments, and control, are shown at Fig. 6.

### GST activity

**Fig. 7:**
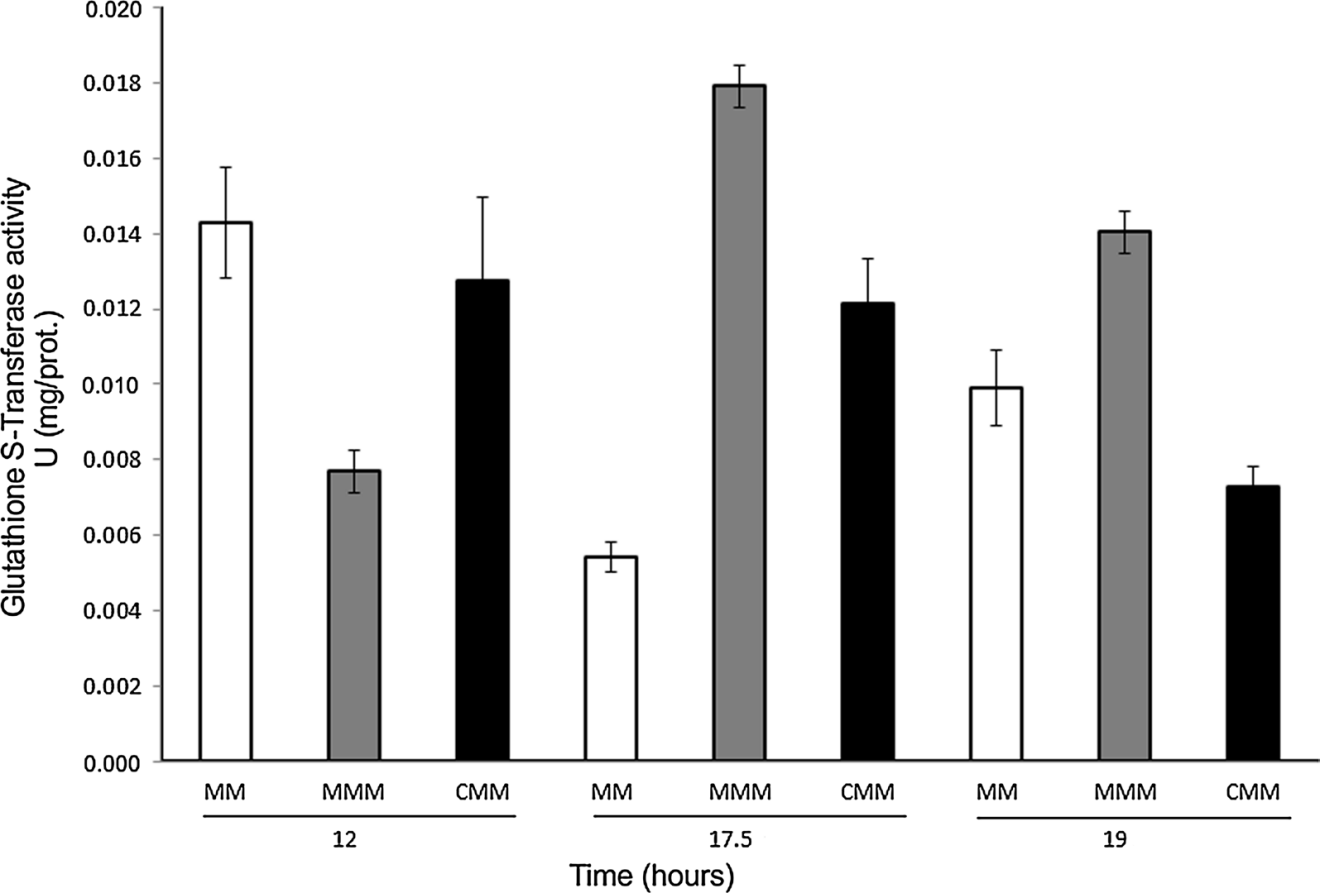
*P. ananatis* CCT 7673 GST activity at 12, 17.5 and 19 h of treatment in the control and in the presence of mesotrione and Callisto^®^. *Error bars* represent standard errors

GST analysis was performed at 12 h (before mesotrione degradation), 17.5 h (immediately before mesotrione degradation) and 19 h (after mesotrione degradation and in the presence of byproducts in the culture medium) (Fig. 7).

### Lipid membrane changes in response to herbicide cytotoxicity

**Fig. 8:**
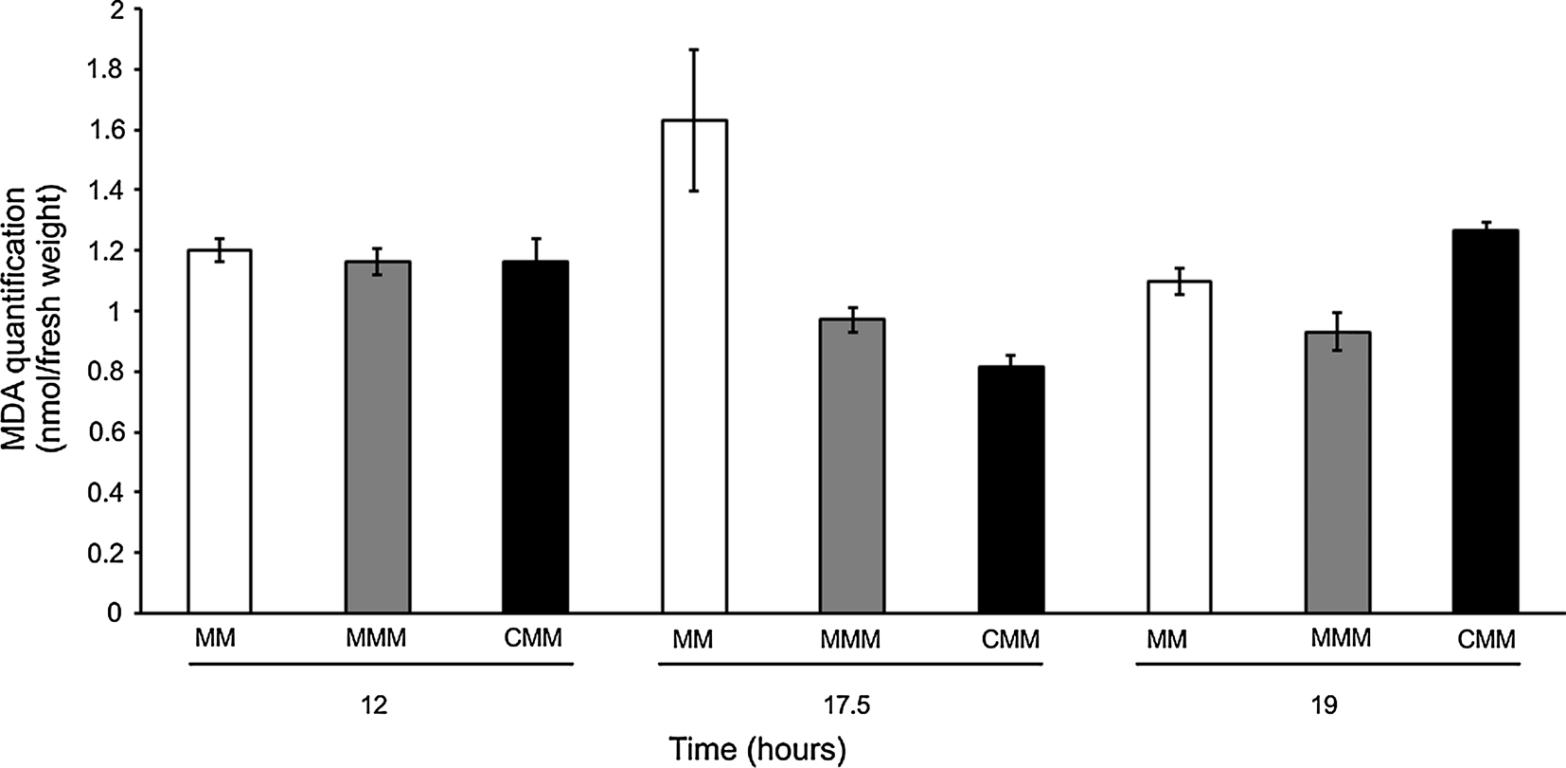
MDA levels (representing lipid peroxidation) in the control-, mesotrione- and Callisto^®^-treated cultures after 12 and 24 h of incubation. *Error bars* represent standard errors

The concentration of MDA was examined in the controls, mesotrione- and Callisto^®^-treated cultures to determine the amount of lipid peroxidation and herbicide cytotoxicity (Fig. 8).

**Fig. 9:**
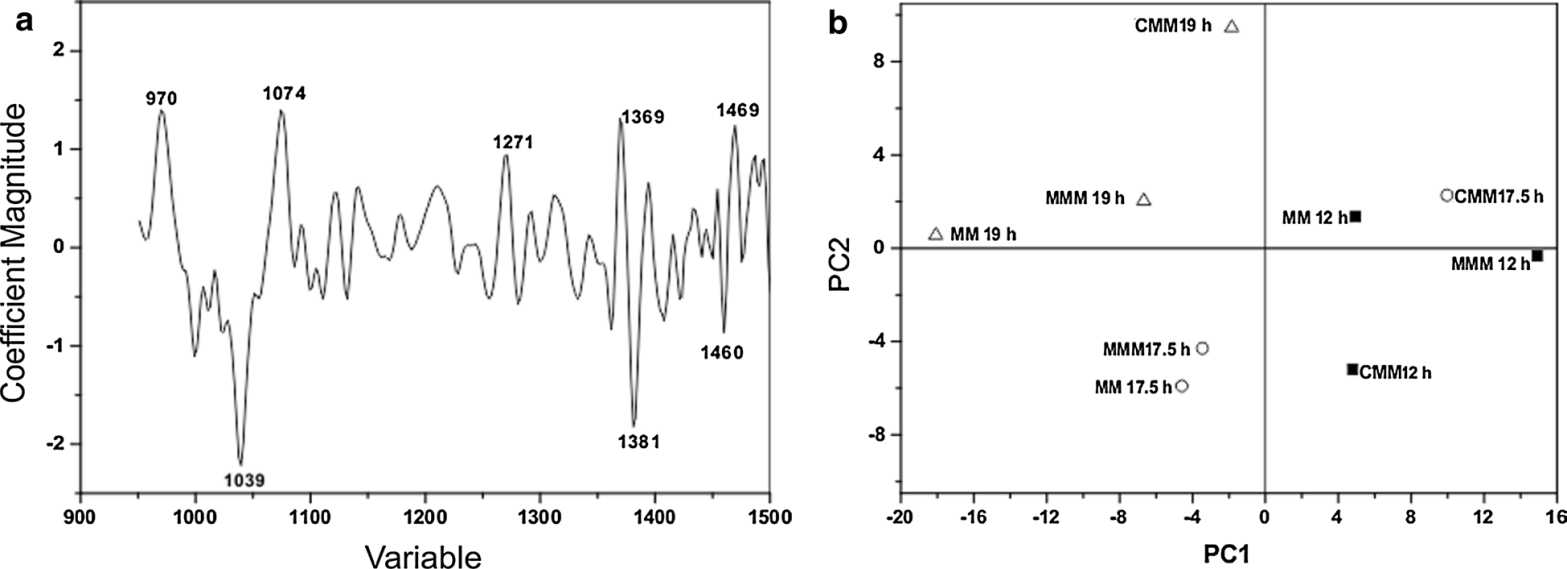
Chemometric analysis of lipids of *P. ananatis*. MM represents the analysis of lipids in the control; MMM are treatments containing mesotrione; and CMM are treatment containing Callisto^®^. Analysis was carried out after 12; 17.5 and 19 h of incubation. The regression vector by PLS and the scores chart by PCA of the data are represented in **a** and **b**, respectively

The lipid saturation data was obtained by FTIR and PCA (Fig. 9a) and PLS (Fig. 9b) statistical analyses. In the regression vector graphic (Fig. 6a), the regions with variables having the highest weight are those that are far from zero and have a higher magnitude coefficient. Positive and negative values indicate directly and inversely proportional relationships between the parameters, respectively. Exploratory PCA and PLS were studied via construction of a matrix with three classes (at 12, 17.5 and 19 h). All spectral regions were evaluated.

## Discussion

### Evaluation of oxidative stress generated by mesotrione

Cellular metabolism in aerobic organisms results in O_2_ reduction and formation of ROS, such as H_2_O_2_ and O_2_^•^. These ROS are generated via normal aerobic metabolic processes of the electron transport chain or due to adverse environmental conditions (Dourado et al. 2015; Gratão et al. 2015), and an increase in ROS can cause oxidative stress in the cell, negatively impacting DNA, RNA and lipids (Gratão et al. 2005). To avoid such damage, organisms possess antioxidant mechanisms that are mediated by enzymes to reduce the impact of ROS, which generally occurs via transcriptional activation of the genes *oxyR* and *soxRS*. ROS-induced damage can be assessed by quantification of H_2_O_2_ in cells, which is one of the main causes of oxidative stress (Gratão et al. 2012), and MDA levels can be used as a marker of oxidative stress (Cabiscol et al. 2000).

The peroxide levels found in this study indicated that mesotrione (p < 0.061, Additional file 1: Section 1.2) and Callisto^®^ (p < 0.015, Additional file 1: Section 1.2) induced oxidative stress in *P. ananatis* (Fig. 2). Moreover, the mesotrione molecule (Additional file 1) contains two groups at its benzoyl positions, 2 and 4, which produce a strong electron-withdrawing effect (Mitchell et al. 2001). These nitro- and methanesulfonyl groups, which are likely responsible for the observed oxidative stress, are also found in MNBA and AMBA, the products of mesotrione degradation by *Bacillus* sp (Durand et al. 2006). In fact, AMBA is more cytotoxic than the active ingredient mesotrione (Mitchell et al. 2001; Bonnet et al. 2008). In contrast, the nitro- and methanesulfonyl groups are removed or modified after 18 h of degradation by *P. ananatis* CCT 7673, producing oxidative stress-generating molecules other than AMBA or MNBA (Pileggi et al. 2012). Although no difference in microbial growth was observed among the treatments and control, the H_2_O_2_ data indicated that mesotrione caused oxidative stress in *P. ananatis.*

### Herbicide tolerance according to bacterial growth

The results of viability and bacterial growth experiments in the MM (control) as well as in MMM and CMM are shown in Figs. 1 and 3, respectively. Viability reached proportional levels in all experiments and controls, compatible with the growth stage. Except at 12 h with mesotrione treatment, before herbicide degradation was complete, in which low viability was found (p < 0.012, Additional file 1: Section 2.1). These analyses suggested that mesotrione decreased the capacity of cell division prior to the time of complete herbicide degradation (18 h). However, this did not diminish overall cell viability, indicating that *P. ananatis* tolerates the herbicide, as previously shown by Pileggi et al. (2012). Therefore, any difference in oxidative stress response in these cultures was likely due to specific induction by the herbicide treatment. Moreover, the elevated H_2_O_2_ levels indicate that the herbicides caused oxidative stress in this bacterium.

### SOD profile

Three SOD bands were found to be active in *P. ananatis* CCT 7673 exposed to the herbicides (Figs. 4, 5), and all bands were more clearly present after 24 h of treatment. The electrophoretic pattern of SOD activity in *P. ananatis* CCT 7673 did not indicate a specific SOD isoenzyme in response to the Callisto^®^/mesotrione treatments.

The Cu/Zn-SOD isoform, indicated by band I, is located within the periplasmic space, but studies of this isoform in this bacterium are scarce; bands II and III correspond to Mn-SOD (Fig. 4), a SOD-type enzyme commonly found in the bacterial cytoplasm that likely responds to increased levels of the O_2_^•^ radical (Shao et al. 2009). The lack of intense change in SOD activity suggested that the treatments did not alter bacterial metabolism because changes in metabolism would result in changes in the production of O_2_^•^. However, the SOD band III showed approximately the same level of activity in all treatments and at all times, whereas the other isoenzymes exhibited some increase in activity, particularly after 24 h.

### CAT activity and gel profiles

The mesotrione molecule is less cytotoxic than Callisto^®^, which is the commercial formulation containing adjuvants (Mitchell et al. 2001; Bonnet et al. 2008). The peroxide rates and levels of CAT activity in *P. ananatis* CCT 7673 at 12 h of treatment supported this hypothesis (Figs. 2, 6). There was increase in CAT activity from 12 to 24 h in control and herbicide-treated cultures. H_2_O_2_ levels may increase 5- 10-fold during the transition from the lag phase to the exponential growth phase in *Escherichia coli* (González-Flecha and Demple 1997). In this study, CAT activity in *P. ananatis* CCT 7673 was dependent on the growth phase (Fig. 3), and the H_2_O_2_ levels increased no more than twofold, probably due the presence of five different isoforms of CAT (Fig. 6). This number of isoforms had not been reported to *P. ananatis* yet. In a previous study with this same bacterial strain, mesotrione was not detectable by HPLC analysis in the culture medium after 24 h (Pileggi et al. 2012), though H_2_O_2_ was produced by the bacterial cells for up to 12 h in response to changes in cell growth as well as to mesotrione and Callisto^®^ (Fig. 2). This production of H_2_O_2_ likely induced higher levels of CAT expression.

### GST activity

With two carboxylic groups with reducing properties, GSH is a non-enzymatic antioxidant that can act as an electron donor and efficiently removes ROS due to its ability to transfer electrons to GST (Ghelfi et al. 2011). GST, in turn, catalyzes the modification of hydrophobic electrolytic substrates, which are normally cytotoxic (Li et al. 2009; Masip and Veeravalli 2006). Furthermore, this enzyme, which is located within the bacterial periplasm, can detoxify xenobiotics (Kullisaar et al. 2010). Pileggi et al. (2012) demonstrated that *P. ananatis* degrades mesotrione after 18 h of incubation. In our study, GST analysis was performed at 12 h (before mesotrione degradation), 17.5 h (immediately before mesotrione degradation) and 19 h (after mesotrione degradation and in the presence of byproducts in the culture medium), and activity was found to be greatest at the last two time points (Fig. 7), corresponding to the mesotrione degradation period in *P. ananatis* CCT 7673. The p value for Bartlett’s Chi square (p = 0.00) suggests that the variances are not homogeneous and that the ANOVA may not be appropriate. So Mann–Whitney/Wilcoxon Two-Sample Test (Kruskal–Wallis test for two groups) was used with p < 0.0180 (Additional file 1: Chapter 3). Therefore, GST is suggested to be involved in mesotrione degradation in this strain. A relationship between GST and herbicide degradation has been observed with several herbicides, such as diuron, oxyfluorfen (Geoffroy 2002), atrazine, chloroacetanilide (Van Eerd et al. 2003) and metolachor (Stamper et al. 2003). However, such a relationship with GST was also found with 2,4-dichlorophenoxyacetic acid (2,4-D) and primisulfuron, two herbicides not known to be metabolized through glutathione conjugation (Pang et al. 2012). Enhancement of the level of free thiol groups has been related to augmented GST activity in different plants (Miteva et al. 2003, 2004). *P. ananatis* CCT 7673 can degrade 100 % of added mesotrione (Pileggi et al. 2012), eventually producing thiol fragments that could be involved in increased GST activity (Fig. 7).

### Lipid membrane changes in response to herbicide cytotoxicity

Malondialdehyde **(**MDA) was examined to determine the amount of lipid peroxidation and herbicide cytotoxicity. As shown in Fig. 8, the MDA values were greater in the controls than mesotrione (p < 0.008, Additional file 1: Section 4.2) and Callisto^®^-treated (p < 0.003, Additional file 1: Section 4.2) cultures mainly at 17.5 h growth phase. It is an unexpected inversion of proportion observed for H_2_O_2_ results (Fig. 2). MDA is a cytotoxic aldehyde product that is released when ROS react with unsaturated fatty acids of the cell membrane, thus causing cell damage (Heath and Packer 1968). Reducing agents, such as glutathione or sulfite, can activate H^+^-ATPases or prevent their inhibition by pro-oxidants, and these effects may be attributable to the location of the H^+^-ATPase in the lipid bilayer (Maeshima 2000). Depending on the stress conditions, the composition of membrane fatty acid residues can prevent lipid peroxidation and MDA production (Ayala et al. 2014). In the presence of oxidants, the stability (mechanical strength) of biological membranes decreases, and antioxidant enzymes may decelerate or inhibit lipid peroxidation reactions, thus enhancing membrane stability. Furthermore, changes in membrane stability, such as those provided by ion channels, can stabilize membranes (Nurminsky et al. 2009).

As MDA serves as an index of peroxidation damage (Heath and Packer 1968), the lipid fraction has been further investigated by mid-infrared spectroscopy to detect changes (Costa Filho 2014). Ogliari et al. (2009) demonstrated that mesotrione promotes differential activation of the primary proton transport system for enzymatic detoxification of this herbicide in maize plants, which can be attributed to changes in lipid conformation after treatment with mesotrione. Freezing and salt stress induce the production of desaturases, which alter unsaturated fatty acids in the membranes of plants and yeasts (Rodríguez-Vargas et al. 2007). Changes in the conformation of saturated and unsaturated fatty acids of the plasma membrane could be considered a protective mechanism by bacteria in contact with herbicides (Murínová and Dercová 2014; Segura et al. 1999). Balague et al. (2001) reported a decrease in unsaturated lipids in the plasma membrane of *E. coli* HB101 after treatment with the herbicide 2,4-D, and these authors considered the reduction in membrane fluidity as a possible defense mechanism against cell damage. Moreover, Sánchez et al. (2005) reported that an increase in the saturation level of membrane lipids in *Klebsiella planticola* DSZ allowed growth in a culture medium containing the herbicide simazine. Similar results were reported by Danilo et al. (1996) for *Ochrobactrum anthropi* cells cultured in the presence of atrazine.

As peroxidation only occurs when lipids are in an unsaturated form, the MDA level depends on the saturation level of lipids (Ayala et al. 2014). Thus, the lower levels of MDA, at log growth phase, compared to the control indicated a change to a higher level of saturation of lipids in *P. ananatis* after treatments with mesotrione and Callisto^®^. Thus, the results suggest changes toward lower membrane permeability, possibly conferring protection against herbicide cytotoxicity.

The hypotheses described above were tested using lipid saturation data obtained by FTIR, and PCA and PLS statistical analyses. PLS relates the variation in incubation time with changes in the FTIR spectrum to assess the functional groups formed or consumed. The regression was performed using the set of FTIR data for those variables (from 950 to 1500 cm^−1^) that showed better discrimination in PCA. The correlation coefficient obtained was 0.93. The others studied variables (Glutathione, GST, H_2_O_2_, MDA, SOD and cell viability) presented lower correlation coefficient values with the FTIR spectrum and therefore were not considered in the analysis of lipid saturation changes.

In the regression vector graphic (Fig. 9a), the regions with variables having the highest weight are those that are far from zero and have a higher magnitude coefficient. Positive and negative values indicate directly and inversely proportional relationships between the parameters, respectively. For example, the variable 1381 cm^−1^ (infrared band) indicates a decrease in the angular deformation of CH bond-plane cis-olefinic groups (unsaturated) with increasing incubation time, possibly increasing membrane permeability. In addition, one phospholipid region at 1085 cm^−1^, a symmetric stretching of the carbonyl (C=O) of COO^−^ groups of fatty acids at 1460 cm^−1^ and acetate groups of saturated alcohols at 1271 cm^−1^ were also identified.

Exploratory PCA and PLS were studied via construction of a matrix with three classes (at 12, 17.5 and 19 h). All spectral regions were evaluated. By applying the preprocessing average centered with multiplicative scatter correction (MSC) and first derived with 99.26 % (PCA) and 99.60 % (PLS) for 7 and 12 principal components (PC), the most informative was between 950.90631 and 1500.6188 cm^−1^, respectively.

PC1 and PC2 in the score chart (Fig. 9a) demonstrated the greatest discrimination among the samples, and differences were related to the unsaturated and saturated fatty acids present. The data indicated that membrane permeability in *P. ananatis* cells after 12 h of incubation is related to unsaturated or saturated/near unsaturated fatty acids in the control and treatments at spectral regions with a number of bands at 1165, 1246, and 1397 cm^−1^. This incubation time represents the log phase of growth (Fig. 1), suggesting that cells require permeable membranes to perform metabolism processes at maximal rates. After 17.5 h of incubation, the band at 1099 cm^−1^ (unsaturated fatty acid) was predominant, characterizing a state of membrane permeability, and this was more conspicuous in MMM medium (Fig. 9b) than in MM medium. According to peroxide production data, mesotrione had a cytotoxic effect on bacterial cells (Fig. 2). In contrast, no CAT- and SOD-specific responses were found at this time. In addition to changes in fatty acid saturation, GST was responsive to mesotrione treatment, with an increase in enzyme activity (Fig. 7) correlated to complete mesotrione degradation at 18 h (Pileggi et al. 2012).

A GST-mesotrione conjugate or induction of GST by free thiol may have been generated during 12–17.5 h of incubation, and such a molecule would need to interact with the cell membrane (Kullisaar et al. 2010) or cytoplasm (Vuilleumier 1997). Pang et al. (2012) demonstrated that glutathione transporters located in the plasma membranes of plants are important components in the glutathione conjugation-related detoxification system. For this reason, the data from the 17.5 h CMM treatment might indicate that the membrane was in a state of impermeability. The herbicide surfactants present in Callisto^®^, 2-(8-methylnonoxy) ethanol and octan-1-ol (https://www3.syngenta.com/country/uk/en/ProductGuide/Pages/Callisto.aspx) are designed to alter the permeability of plant cells. Moreover, 1,2-benzisothiazolin-3-one (https://www.archive.epa.gov/pesticides/reregistration/web/pdf/benzisothiazolin_red.pdf) has an antimicrobial function, which likely helps to prevent microbial degradation of mesotrione. PCA and PLS analyses of the 17.5 h CMM treatment suggested that Callisto^®^ adjuvants interfered with and changed the structure of the *P. ananatis* cytoplasmic membrane (Mesnage et al. 2013).

At the 19 h time point, the presence of the band at 1240 cm^−1^ (Silverstein and Webster 1998) indicated that *P. ananatis* membrane impermeability increased in the MMM treatment and even more so in CMM.

In general, an increase in saturated fatty acids in *P. ananatis* was observed in response to mesotrione treatment, with a greater increase in Callisto^®^-treated cells (Fig. 9). These data appear to be correlated to a decrease in membrane permeability, as a response to the cytotoxicity of the herbicide and adjuvants. Despite the increase in H_2_O_2_ (Fig. 2), the decrease in MDA concentration in the mesotrione and Callisto^®^ treatments (Fig. 8) corroborated this hypothesis because the MDA measured originated from the peroxidation of unsaturated fatty acids.

The commercial formulation of the Callisto^®^ herbicide, and its active ingredient mesotrione at concentrations that are commonly used in the environment, cause oxidative stress in *P. ananatis* CCT 7673 through increased H_2_O_2_ production and greater GST enzyme expression, which is likely involved in herbicide degradation. Despite this, the bacterium can tolerate and grow in the presence of the herbicides. No CAT- and SOD-specific responses were observed to explain this herbicide tolerance, and the bacterium exhibited low amounts of MDA, an index of peroxidation damage, under herbicide treatments. A pattern change to a higher level of lipid saturation was suggested by PCA and PLS analyses, possibly conferring a protective effect to bacteria cells through reduced bacterial membrane permeability. In the mesotrione treatments, this increase was likely related to the formation of a GST-mesotrione conjugated adduct. Changes in membrane fatty acid saturation in the Callisto^®^ treatments were of a greater degree than in the mesotrione treatments. These events were likely related to the adjuvant content, which interfered with the structure of the lipid membrane. Taken together, these features make *P. ananatis* an excellent model for studying herbicide tolerance in both soil and water environments.

## Additional file

10.1186/s13568-016-0240-x Mesotrione molecular structure and statistical analysis of peroxide, viabilty, GST and MDA data.

## Abbreviations

GST: glutathione-S-transferase
MDA: malondialdehyde
HPPD: 4-hydroxyphenylpyruvate dioxygenase
MNBA: 4-methylsulfonyl-2-nitrobenzoic acid
AMBA: 2-amino-4-methylsulfonyl benzoic acid
ROS: reactive oxygen species
H_2_O_2_: hydrogen peroxide
O_2_^•^: superoxide radical
OH^•^: hydroxyl radical
HPLC: high-performance liquid chromatography
LB: Luria Broth
PBS: phosphate-buffered saline
MM: mineral medium
MMM: mesotrione mineral medium
CMM: callisto mineral medium
LA: luria agar
TCA: tricloroacetic acid
TBA: thiobarbituric acid
FTIR: Fourier transformed infrared spectroscopy
PCA: principal component analysis
PLS: partial least squares
EDTA: ethylenediaminetetraacetic acid
BSA: bovine serum albumin
PAGE: polyacrylamide gel electrophoresis
SDS: sodium dodecyl sulfate
SOD: superoxide dismutase
CAT: catalase
GSH: glutathione sulfhydryl
CDNB: 1-cloro-2,4-dinitrobenzene
DCNB: 1, 2-dichloro-4-nitrobenzene

## References

[CR1] Ayala A, Muñoz MF, Argüelles S (2014). Lipid peroxidation: production, metabolism, and signaling mechanisms of malondialdehyde and 4-hydroxy-2-nonenal. Oxid Med Cell Longevity.

[CR2] Azevedo RA, Alas RM, Smith RJ, Lea PJ (1998). Response of antioxidant enzymes to transfer from elevated carbon dioxide to air and ozone fumigation, in the leaves and roots of wild-type and a catalase-deficient mutant of barley. Physiol Plant.

[CR3] Balague C, Sturtz N, Duffard R, De Duffard AME (2001). Effect of 2,4-dichlorophenoxyacetic acid herbicide on *Escherichia coli* growth, chemical composition and cellular envelope. Environ Toxicol.

[CR4] Batisson I, Crouzet O, Besse-Hoggan P, Sancelme M, Mangot J, Mallet C, Bohatier J (2009). Isolation and characterization of mesotrione—degrading *Bacillus* sp. from soil. Environ Pollut.

[CR5] Bjørling-Poulsen M, Andersen HR, Grandjean P (2008). Potential developmental neurotoxicity of pesticides used in Europe. Environ Health.

[CR6] Bligh E, Dyer G, Justin W (1959). A rapid method of total lipid extraction and purification. Can J Biochem Physiol.

[CR7] Boaretto LF, Carvalho G, Borgo L, Creste S, Landell MGA, Mazzafera P, Azevedo RA (2014). Water stress reveals differential antioxidant responses of tolerant and non-tolerant sugarcane genotypes. Plant Physiol Biochem.

[CR8] Bonnet JL, Bonnemoy F, Dusser M, Bohatier J (2008). Toxicity assessment of the herbicides Sulcotrione and Mesotrione toward two reference environmental micro-organisms: *Tetrahymena pyriformis* and *Vibrio fischeri*. Arch Environ Contam Toxicol.

[CR9] Bradford MM (1976). A rapid and sensitive method for the quantification of microgram quantities of protein utilizing the principle of protein-dye binding. Anal Biochem.

[CR10] Cabiscol E, Tamarit J, Ros J (2000). Oxidative stress in bacteria and protein damage by reactive oxygen species. Int Microbiol.

[CR11] Copley SD (2009). Evolution of efficient pathways for degradation of anthropogenic chemicals. Nat Chem Biol.

[CR12] Costa Filho PA (2014). Developing a rapid and sensitive method for determination of trans-fatty acids in edible oils using middle-infrared spectroscopy. Food Chem.

[CR13] Crouzet O, Batisson I, Besse-Hoggan P, Bonnemoy F, Bardot C, Poly F, Bohatier J, Mallet C (2010). Response of soil microbial communities to the herbicide mesotrione: a dose-effect microcosm approach. Soil Biol Biochem.

[CR14] Danilo L, Giacomo S, Frassanito R, Rotillo D (1996). Effects of atrazine on *Ochrobactrum anthropi* membrane fatty acids. Appl Environ Microbiol.

[CR15] Dourado MN, Franco MR, Peters LP, Martins PF, Souza LA, Piotto FA, Azevedo RA (2015). Antioxidant enzymes activities of *Burkholderia* spp. strains—oxidative responses to Ni toxicity. Environ Sci Pollut Res.

[CR16] Dourado MN, Martins PF, Quecine MC, Piotto FA, Souza LA, Franco MR, Tezotto T, Azevedo RA (2013). *Burkholderia* sp. SCMS54 reduces cadmium toxicity and promotes growth in tomato. Ann Appl Biol.

[CR17] Durand S, Amato P, Sancelme M, Delort AM, Combourieu B, Besse-Hoggan P (2006). First isolation and characterization of a bacterial strain that biotransforms the herbicide mesotrione. Lett Appl Microbiol.

[CR18] Geoffroy L (2002). Effect of oxyfluorfen and diuron alone and in mixture on antioxidative enzymes of *Scenedesmus obliquos*. Pest Biochem Physiol.

[CR19] Ghelfi A, Gaziola SA, Cia MC, Chabregas SM, Falco MC, Kuser-Falcão PR, Azevedo RA (2011). Cloning, expression, molecular modelling and docking analysis of glutathione transferase from *Saccharum officinarum*. Ann Appl Biol.

[CR20] González-Flecha B, Demple B (1997). Metabolic sources of hydrogen-peroxide in aerobically growing *Escherichia coli*. J Biol Chem.

[CR21] Gratão PL, Monteiro CC, Carvalho RF, Tezotto T, Piotto FA, Peres LEP, Azevedo RA (2012). Biochemical dissection of diageotropica and Never ripe tomato mutants to Cd-stressful conditions. Plant Physiol Biochem.

[CR22] Gratão PL, Monteiro CC, Tezotto T, Carvalho RF, Alves LR, Peters LP, Azevedo RA (2015). Cadmium stress antioxidant responses and root-to-shoot communication in grafted tomato plants. Biometals.

[CR23] Gratão PL, Polle A, Lea PJ, Azevedo RA (2005). Making the life of heavy metal-stressed plants a little easier. Func Plant Biol.

[CR24] Heath RL, Packer L (1968). Photoperoxidation in isolated chloroplasts. I. Kinetics and stoichiometry of fatty acid peroxidation. Arch Biochem Biophys.

[CR25] Kullisaar T, Songisepp E, Aunapuu M, Kilk K, Arend A, Mikelsaar M, Rehema A, Zilmer M (2010). Complete Gluthaione system in Probiotic *Lactobacillus fermentum* ME-3. App Biochem Microbiol.

[CR26] Li Z, Shao T, Min H, Lu Z, Xu X (2009). Stress response of *Burkholderia cepacia* WZ1 exposed to quinclorac and the biodegradation of quinclorac. Soil Biol Biochem.

[CR27] Lushchak VI (2001). Oxidative stress and mechanisms of protection against it in bacteria. Biochemistry.

[CR28] Maeshima M (2000). Vacuolar H^+^-pyrophosphatase. Biochim Biophys Acta.

[CR29] Martins PF, Carvalho G, Gratão PL, Dourado MN, Pileggi M, Araújo WL, Azevedo RA (2011). Effects of the herbicides acetochlor and metolachlor on antioxidant enzymes in soil bacteria. Process Biochem.

[CR30] Martins PF, Martinez CO, Carvalho G, Carneiro PIB, Azevedo RA, Pileggi SAV, Melo IS, Pileggi M (2007). Selection of microorganisms degrading S-metolachlor herbicide. Braz Arch Biol Technol.

[CR31] Masip L, Veeravalli K (2006). The many faces of glutathione in bacteria. Antioxid Redox Signal.

[CR32] Mesnage R, Bernay B, Séralini G-E (2013). Ethoxylated adjuvants of glyphosate-based herbicides are active principles of human cell toxicity. Toxicol.

[CR33] Mitchell G, Bartlett DW, Fraser TEM, Hawkes TR, Holt DC, Townson JK, Wichert RA (2001). Mesotrione: a new selective herbicide for use in maize. Pest Manag Sci.

[CR34] Miteva L, Ivanov S, Alexieva V, Karanov E (2003). Effect of herbicide glyphosate on glutathione levels, glutathione s-transferase and glutathione reductase activities in two plant species. Biologie Physiolgie des Plantes.

[CR35] Miteva LP-E, Ivanov SV, Alexieva VS, Karanov EN (2004). Effect of atrazine on glutathione levels, glutathione s-transferase and glutathione reductase activities in pea and wheat plants. Plant Protect Sci.

[CR36] Monteiro CC, Carvalho RF, Gratão PL, Carvalho G, Tezotto T, Medici LO, Peres LEP, Azevedo RA (2011). Biochemical responses of the ethylene insensitive Never ripe tomato mutant subjected to cadmium and sodium stresses. Environ Exp Bot.

[CR37] Murínová S, Dercová K (2014). Response mechanisms of bacterial degraders to environmental contaminants on the level of cell walls and cytoplasmic membrane. Inter J Microbiol.

[CR38] Nurminsky VN, Ozolina NV, Sapega JG, Zheleznykh AO, Pradedova EV, Korzun AM, Salyaev RK (2009). The effect of dihydroquercetin on active and passive ion transport systems in plant vacuolar membrane. Biol Bull.

[CR39] Ogliari J, Freitas SP, Ramos AC, Bressan-Smith RE, Façanha AR (2009). Proton transport primary systems used as mechanisms of mesotrione detoxification in corn plants. Planta Daninha.

[CR40] Olchanheski LR, Dourado MN, Beltrame FL, Zielinski AAF, Demiate IM, Pileggi SAV, Azevedo RA, Sadowsky MJ, Pileggi M (2014). Mechanisms of tolerance and high degradation capacity of the herbicide mesotrione by *Escherichia coli* strain DH5-a. PLoS ONE.

[CR41] Pang S, Duan L, Liu Z, Song X, Li X, Wang C (2012). Co-Induction of a glutathione-S-transferase, a glutathione transporter and an ABC transporter in maize by xenobiotics. PLoS ONE.

[CR42] Peters LP, Carvalho G, Martins PF, Dourado MN, Vilhena MB, Pileggi M, Azevedo RA (2014). Differential responses of the antioxidant system of ametryn and clomazone tolerant bacteria. PLoS ONE.

[CR43] Pileggi M, Pileggi SAV, Olchanheski LR, Silva PAG, Gonzalez AMM, Koskinen WC, Barber B, Sadowsky MJ (2012). Isolation of mesotrione-degrading bacteria from aquatic environments in Brazil. Chemosphere.

[CR44] Rodríguez-Vargas S, Sánchez-García A, Martínez-Rivas JM, Prieto JA, Randez-Gil F (2007). Fluidization of membrane lipids enhances the tolerance of Saccharomyces cerevisiae to freezing and salt stress. Appl Environ Microbiol.

[CR45] Sánchez M, Garbi C, Martínez-Álvarez R, Ortiz LT, Allende JL, Martín M (2005). *Klebsiella planticola* strain DSZ mineralizes simazine: physiological adaptations involved in the process. Appl Microbiol Biotechnol.

[CR46] Segura A, Duque E, Mosqueda G, Ramos JL, Junker F (1999). Multiple responses of Gram-negative bacteria to organic solvents. Environ Microbiol.

[CR47] Shao T, Yuan H, Yan B, Lü Z, Min H (2009). Antioxidant enzyme activity in bacterial resistance to nicotine toxicity by reactive oxygen species. Arch Environ Contam Toxicol.

[CR48] Silverstein RM, Webster FX (1998). Spectrometric identification of organic compounds.

[CR49] Simon C, Daniel R (2011). Metagenomic analyses: past and future trends. Appl Environ Microbiol.

[CR50] Stamper D, Popovic M, Stajner M (2003). Herbicide induced oxidative stress in lettuce, beans, pea seeds and leaves. Biol Plant.

[CR51] Stoob K, Singer HP, Goetz CW, Ruff M, Mueller SR (2005). Fully automated online solid phase extraction coupled directly to liquid chromatography—tandem mass spectrometry quantification of sulfonamide antibiotics, neutral and acidic pesticides at low concentrations in surface waters. J Chromatogr A.

[CR52] Tétard-Jones C, Robert E (2015). Potential roles for microbial endophytes in herbicide tolerance in plants. Pest Manag Sci.

[CR53] Van Eerd LL, Hoagland RE, Zablotowicz RM, Hall JC (2003). Pesticides metabolism in plants and microorganisms. Weed Sci.

[CR54] Vuilleumier S (1997). Bacterial glutathione S-transferases: what are they good for?. J Bacteriol.

