# Supplementary material for: GST activity and membrane lipid saturation prevents mesotrione-induced cellular damage in Pantoea ananatis
Source: AMB Express. 2016 Sep 13;6(1):70. doi: 10.1186/s13568-016-0240-x (PMC5020000; doi:10.1186/s13568-016-0240-x)
Supplement: Supplementary file 1 — 10.1186/s13568-016-0240-x Mesotrione molecular structure and statistical analysis of peroxide, viabilty, GST and MDA data. [file 13568_2016_240_MOESM1_ESM.docx]

**AMB Express**

**GST Activity and Membrane Lipid Saturation Prevents Mesotrione-induced Cellular Damage**

**in *Pantoea ananatis***

Lilian P. Prione^1^, Luiz R. Olchanheski^2^, Leandro D. Tullio^1^, Bruno C.E. Santo^1^, Péricles M. Reche^3^, Paula F. Martins^4^, Giselle Carvalho^4^, Ivo M. Demiate^5^, Sônia A.V. Pileggi^1^, Manuella N. Dourado^2^, Rosilene A. Prestes^6^, Michael J. Sadowsky^7^, Ricardo A. Azevedo^4^, and Marcos Pileggi^1*^

^1^ Departamento de Biologia Estrutural, Molecular e Genética, Universidade Estadual de Ponta Grossa, Campus Universitário de Uvaranas, Av. Carlos Cavalcanti, 4748, 84030-900, Ponta Grossa, Paraná, Brazil.

^2^ Departamento de Microbiologia, Instituto de Ciências Biomédicas, Universidade de São Paulo, 05508-000, São Paulo, São Paulo, Brazil.

^3^ Departamento de Enfermagem e Saúde Pública, Universidade Estadual de Ponta Grossa, Campus Universitário de Uvaranas, Av. Carlos Cavalcanti, 4748, 84030-900, Ponta Grossa, Paraná, Brazil.

^4^ Departamento de Genética, Escola Superior de Agricultura Luiz de Queiroz, Universidade de São Paulo, Piracicaba, São Paulo, Brazil.

^5^ Universidade Estadual de Ponta Grossa, UEPG, Departamento de Engenharia de Alimentos, Av. Carlos Cavalcanti, 4748, 84030-900, Ponta Grossa, PR, Brazil.

^6^ Universidade Tecnológica Federal do Paraná, UTFPR, Campus Ponta Grossa, Av. Monteiro Lobato, 84016-210, Ponta Grossa, PR, Brazil.

^7^ Department of Soil, Water, and Climate, and The BioTechnology Institute, University of Minnesota, St. Paul, Minnesota, MN 55108, USA.

***** e-mail: pileggimarcos@gmail.com

Phone number: 55 42 3220 3737

Fax number: 55 42 3220 3102





Mesotrione molecular structure.

**Statistical Analysis**

Chapter 1 - H_2_O_2_

Section 1.1 - 12 hours treatments,bonferroni means st

| Summary of 12 hours

treatmens | Mean Std. Dev.

------------+------------------------

MM | 1.569402 .50894843

MMM | 1.133457 .24431367

CMM | 1.5258075 .64791588

------------+------------------------

Total | 1.4095555 .49329331

Analysis of Variance

Source SS df MS F Prob > F

------------------------------------------------------------------------

Between groups .461183219 2 .230591609 0.94 0.4270

Within groups 2.21553798 9 .246170886

------------------------------------------------------------------------

Total 2.67672119 11 .24333829

Comparison of 12 hours by treatments

(Bonferroni)

Row Mean-|

Col Mean | MM MMM

---------+----------------------

MMM | -.435945

| 0.736

|

CMM | -.043595 .39235

| 1.000 0.877

Section 1.2 - 17.5 hours treatments,bonferroni means st

| Summary of 17.5 hours

treatmens | Mean Std. Dev.

------------+------------------------

mm 1 | .69859199 .10083306

mmm 2 | 1.52817 .3537151

mmc 3 | 1.804696 .49626077

------------+------------------------

Total | 1.4024764 .57716373

Analysis of Variance

Source SS df MS F Prob > F

------------------------------------------------------------------------

Between groups 2.19667772 2 1.09833886 7.74 0.0135

Within groups 1.134502 8 .14181275

------------------------------------------------------------------------

Total 3.33117972 10 .333117972

Bartlett's test for equal variances: chi2(2) = 3.4003 Prob>chi2 = 0.183

Comparison of 17.5 by treatments

(Bonferroni)

Row Mean-|

Col Mean | MM MMM

---------+----------------------

MMM | .829578

| 0.061

|

CMM | 1.1061 .276526

| 0.015 0.988

Section 1.3 - 19 hours treatments_3,bonferroni means st

| Summary of 19 hours

treatments | Mean Std. Dev.

------------+------------------------

MM | .69859201 .20719226

MMM | 2.1151814 .34111462

CMM | 2.1151814 .34111462

------------+------------------------

Total | 1.5485456 .77533961

Analysis of Variance

Source SS df MS F Prob > F

------------------------------------------------------------------------

Between groups 4.81614099 2 2.4080705 28.37 0.0004

Within groups .594222645 7 .084888949

------------------------------------------------------------------------

Total 5.41036364 9 .601151515

Bartlett's test for equal variances: chi2(2) = 0.6543 Prob>chi2 = 0.721

Comparison of 19 hours by treatments

(Bonferroni)

Row Mean-|

Col Mean | MM MMM

---------+----------------------

MMM | 1.41659

| 0.001

|

CMM | 1.41659 0

| 0.001 1.000

Section 1.4 - mm12xmmm hours, bonferroni means st

| Summary of mm12 x mmm

| 17.5 x mmm19

hours | Mean Std. Dev.

------------+------------------------

12 | 1.569402 .50894843

17.5 | 1.52817 .3537151

19 | 2.1151814 .34111462

------------+------------------------

Total | 1.7032575 .45699918

Analysis of Variance

Source SS df MS F Prob > F

------------------------------------------------------------------------

Between groups .70333553 2 .351667765 2.03 0.1935

Within groups 1.385147 8 .173143375

------------------------------------------------------------------------

Total 2.08848253 10 .208848253

Bartlett's test for equal variances: chi2(2) = 0.4781 Prob>chi2 = 0.787

Comparison of mm12 x mmm17.5 x mmm19 by hours

(Bonferroni)

Row Mean-|

Col Mean | 12 17.5

---------+----------------------

17.5 | -.041232

| 1.000

|

19 | .545779 .587011

| 0.373 0.306

Section 1.5 - mm17xmmm hours, bonferroni means st

| Summary of mmm12 x

| mm17.5 x mmm19

hours | Mean Std. Dev.

------------+------------------------

12 | 1.133457 .24431367

17.5 | .69859199 .10083306

19 | 2.1151814 .34111462

------------+------------------------

Total | 1.2975148 .63435372

Analysis of Variance

Source SS df MS F Prob > F

------------------------------------------------------------------------

Between groups 3.18952127 2 1.59476063 25.83 0.0006

Within groups .432120499 7 .0617315

------------------------------------------------------------------------

Total 3.62164177 9 .402404641

Bartlett's test for equal variances: chi2(2) = 2.0364 Prob>chi2 = 0.361

Comparison of mmm12 x mm17.5 x mmm19 by hours

(Bonferroni)

Row Mean-|

Col Mean | 12 17.5

---------+----------------------

17.5 | -.434865

| 0.167

|

19 | .981724 1.41659

| 0.004 0.001

Section 1.6 - mm19xmmm hours, bonferroni means st

| Summary of mmm12 x

| mmm17.5 x mm19

hours | Mean Std. Dev.

------------+------------------------

12 | 1.133457 .24431367

17.5 | 1.52817 .3537151

19 | .69859201 .20719226

------------+------------------------

Total | 1.120073 .43282062

Analysis of Variance

Source SS df MS F Prob > F

------------------------------------------------------------------------

Between groups 1.37747405 2 .688737024 9.07 0.0070

Within groups .683196532 9 .075910726

------------------------------------------------------------------------

Total 2.06067058 11 .187333689

Bartlett's test for equal variances: chi2(2) = 0.8120 Prob>chi2 = 0.666

Comparison of mmm12 x mmm17.5 x mm19 by hours

(Bonferroni)

Row Mean-|

Col Mean | 12 17.5

---------+----------------------

17.5 | .394713

| 0.220

|

19 | -.434865 -.829578

| 0.158 0.006

Section 1.7 - mm12xcmm hours, bonferroni means st

| Summary of mm12 x

| cmm17.5 x cmm19

hours | Mean Std. Dev.

------------+------------------------

12 | 1.569402 .50894843

17.5 | 1.804696 .49626077

19 | 2.1151814 .34111462

------------+------------------------

Total | 1.8038124 .47531843

Analysis of Variance

Source SS df MS F Prob > F

------------------------------------------------------------------------

Between groups .510647948 2 .255323974 1.17 0.3588

Within groups 1.74862814 8 .218578518

------------------------------------------------------------------------

Total 2.25927609 10 .225927609

Bartlett's test for equal variances: chi2(2) = 0.3354 Prob>chi2 = 0.846

Comparison of mm12 x cmm17.5 x cmm19 by hours

(Bonferroni)

Row Mean-|

Col Mean | 12 17.5

---------+----------------------

17.5 | .235294

| 1.000

|

19 | .545779 .310485

| 0.495 1.000

Section 1.8 - mm17xcmm hours, bonferroni means st

| Summary of cmm12 x

| mm17.5 x cmm19

hours | Mean Std. Dev.

------------+------------------------

12 | 1.5258075 .64791588

17.5 | .69859199 .10083306

19 | 2.1151814 .34111462

------------+------------------------

Total | 1.454455 .71152944

Analysis of Variance

Source SS df MS F Prob > F

------------------------------------------------------------------------

Between groups 3.04402939 2 1.5220147 7.04 0.0211

Within groups 1.51243795 7 .216062564

------------------------------------------------------------------------

Total 4.55646734 9 .506274149

Bartlett's test for equal variances: chi2(2) = 4.4709 Prob>chi2 = 0.107

Comparison of cmm12 x mm17.5 x cmm19 by hours

(Bonferroni)

Row Mean-|

Col Mean | 12 17.5

---------+----------------------

17.5 | -.827216

| 0.158

|

19 | .589374 1.41659

| 0.423 0.022

Section 1.9 - mm19xcmm hours, bonferroni means st

| Summary of cmm12 x

| cmm17.5 x mm19

hours | Mean Std. Dev.

------------+------------------------

12 | 1.5258075 .64791588

17.5 | 1.804696 .49626077

19 | .69859201 .20719226

------------+------------------------

Total | 1.3430318 .65881221

Analysis of Variance

Source SS df MS F Prob > F

------------------------------------------------------------------------

Between groups 2.64737372 2 1.32368686 5.60 0.0263

Within groups 2.12699512 9 .236332791

------------------------------------------------------------------------

Total 4.77436884 11 .434033531

Bartlett's test for equal variances: chi2(2) = 2.8480 Prob>chi2 = 0.241

Comparison of cmm12 x cmm17.5 x mm19 by hours

(Bonferroni)

Row Mean-|

Col Mean | 12 17.5

---------+----------------------

17.5 | .278888

| 1.000

|

19 | -.827215 -1.1061

| 0.118 0.032

Chapter 2 – Viability

Section 2.1 | Summary of 12 hours

treatments | Mean Std. Dev.

------------+------------------------

mm 1 | 4.615e+10 6.178e+09

mmm 2 | 2.815e+10 4.547e+09

cmm 3 | 4.760e+10 4.980e+09

------------+------------------------

Total | 4.063e+10 1.044e+10

Analysis of Variance

Source SS df MS F Prob > F

------------------------------------------------------------------------

Between groups 7.0440e+20 2 3.5220e+20 12.63 0.0071

Within groups 1.6727e+20 6 2.7879e+19

------------------------------------------------------------------------

Total 8.7168e+20 8 1.0896e+20

Bartlett's test for equal variances: chi2(2) = 0.1671 Prob>chi2 = 0.920

Comparison of mmc12 by viablcod

(Bonferroni)

Row Mean-|

Col Mean | MM MMM

---------+----------------------

MMM | -1.8e+10

| 0.018

|

CMM | 1.5e+09 1.9e+10

| 1.000 0.012

Section 2.2 - 17.5 hours treatments, bonferroni means st

| Summary of 17.5 hours

treatmens | Mean Std. Dev.

------------+------------------------

MM | 4.767e+10 3.924e+09

MMM | 1.507e+11 1.703e+11

CMM | 5.347e+10 1.537e+10

------------+------------------------

Total | 8.395e+10 9.915e+10

Analysis of Variance

Source SS df MS F Prob > F

------------------------------------------------------------------------

Between groups 2.0111e+22 2 1.0055e+22 1.03 0.4123

Within groups 5.8533e+22 6 9.7555e+21

------------------------------------------------------------------------

Total 7.8643e+22 8 9.8304e+21

Bartlett's test for equal variances: chi2(2) = 14.8611 Prob>chi2 = 0.001

Comparison of 17.5 hours by treatments

(Bonferroni)

Row Mean-|

Col Mean | MM MMM

---------+----------------------

MMM | 1.0e+11

| 0.746

|

CMM | 5.8e+09 -9.7e+10

| 1.000 0.820

.

Section 2.3 - 19 hours treatments, bonferroni means st

| Summary of 19 hours

treatments | Mean Std. Dev.

------------+------------------------

MM | 2.798e+10 7.286e+08

MMM | 2.645e+10 1.182e+09

CMM | 2.840e+10 1.622e+09

------------+------------------------

Total | 2.761e+10 1.390e+09

Analysis of Variance

Source SS df MS F Prob > F

------------------------------------------------------------------------

Between groups 6.3272e+18 2 3.1636e+18 2.08 0.2058

Within groups 9.1217e+18 6 1.5203e+18

------------------------------------------------------------------------

Total 1.5449e+19 8 1.9311e+18

Bartlett's test for equal variances: chi2(2) = 0.9611 Prob>chi2 = 0.618

Comparison of 19 hours by treatments

(Bonferroni)

Row Mean-|

Col Mean | MM MMM

---------+----------------------

MMM | -1.5e+09

| 0.536

|

CMM | 4.2e+08 2.0e+09

| 1.000 0.303

Section 2.4 - viabmm0.5xmmm hours, bonferroni means st

| Summary of mm0.5 x mmm12

| x mmm17.5 x mmm19

hours | Mean Std. Dev.

------------+------------------------

0.5 | 3.617e+08 27537853

12 | 2.815e+10 4.547e+09

17.5 | 1.507e+11 1.703e+11

19 | 2.645e+10 1.182e+09

------------+------------------------

Total | 5.142e+10 9.484e+10

Analysis of Variance

Source SS df MS F Prob > F

------------------------------------------------------------------------

Between groups 4.0885e+22 3 1.3628e+22 1.88 0.2117

Within groups 5.8054e+22 8 7.2567e+21

------------------------------------------------------------------------

Total 9.8939e+22 11 8.9945e+21

Bartlett's test for equal variances: chi2(3) = 48.1728 Prob>chi2 = 0.000

Section 2.5 - Comparison of mm0.5 x mmm12 x mmm17.5 x mmm19 by hours

(Bonferroni)

Row Mean-|

Col Mean | 0.5 12 17.5

---------+---------------------------------

12 | 2.8e+10

| 1.000

|

17.5 | 1.5e+11 1.2e+11

| 0.376 0.697

|

19 | 2.6e+10 -1.7e+09 -1.2e+11

| 1.000 1.000 0.671

viabmm12xmmm hours, bonferroni means st

| Summary of mmm0.5 x mm12

| x mmm17.5 x mmm19

hours | Mean Std. Dev.

------------+------------------------

0.5 | 4.250e+08 26457513

12 | 4.615e+10 6.178e+09

17.5 | 1.507e+11 1.703e+11

19 | 2.645e+10 1.182e+09

------------+------------------------

Total | 5.593e+10 9.399e+10

Analysis of Variance

Source SS df MS F Prob > F

------------------------------------------------------------------------

Between groups 3.9081e+22 3 1.3027e+22 1.79 0.2261

Within groups 5.8089e+22 8 7.2611e+21

------------------------------------------------------------------------

Total 9.7169e+22 11 8.8336e+21

Bartlett's test for equal variances: chi2(3) = 47.2944 Prob>chi2 = 0.000

Section 2.6 - Comparison of mmm0.5 x mm12 x mmm17.5 x mmm19 by hours

(Bonferroni)

Row Mean-|

Col Mean | 0.5 12 17.5

---------+---------------------------------

12 | 4.6e+10

| 1.000

|

17.5 | 1.5e+11 1.0e+11

| 0.377 1.000

|

19 | 2.6e+10 -2.0e+10 -1.2e+11

| 1.000 1.000 0.672

Section 2.7 - viabmm17.5xmmm hours, bonferroni means st

| Summary of mmm0.5 x mmm12

| x mm17.5 x mmm19

hours | Mean Std. Dev.

------------+------------------------

0.5 | 4.250e+08 26457513

12 | 2.815e+10 4.547e+09

17.5 | 4.767e+10 3.924e+09

19 | 2.645e+10 1.182e+09

------------+------------------------

Total | 2.567e+10 1.773e+10

Analysis of Variance

Source SS df MS F Prob > F

------------------------------------------------------------------------

Between groups 3.3838e+21 3 1.1279e+21 120.42 0.0000

Within groups 7.4933e+19 8 9.3666e+18

------------------------------------------------------------------------

Total 3.4587e+21 11 3.1443e+20

Bartlett's test for equal variances: chi2(3) = 16.7428 Prob>chi2 = 0.001

Section 2.7 - Comparison of mmm0.5 x mmm12 x mm17.5 x mmm19 by hours

(Bonferroni)

Row Mean-|

Col Mean | 0.5 12 17.5

---------+---------------------------------

12 | 2.8e+10

| 0.000

|

17.5 | 4.7e+10 2.0e+10

| 0.000 0.000

|

19 | 2.6e+10 -1.7e+09 -2.1e+10

| 0.000 1.000 0.000

viabmm19xmmm hours, bonferroni means st

| Summary of mmm0.5 x mmm12

| x mmm17.5 x mm19

hours | Mean Std. Dev.

------------+------------------------

0.5 | 4.250e+08 26457513

12 | 2.815e+10 4.547e+09

17.5 | 1.507e+11 1.703e+11

19 | 2.798e+10 7.286e+08

------------+------------------------

Total | 5.181e+10 9.472e+10

Analysis of Variance

Source SS df MS F Prob > F

------------------------------------------------------------------------

Between groups 4.0641e+22 3 1.3547e+22 1.87 0.2135

Within groups 5.8052e+22 8 7.2565e+21

------------------------------------------------------------------------

Total 9.8693e+22 11 8.9721e+21

Bartlett's test for equal variances: chi2(3) = 49.9073 Prob>chi2 = 0.000

Section 2.8 - Comparison of mmm0.5 x mmm12 x mmm17.5 x mm19 by hours

(Bonferroni)

Row Mean-|

Col Mean | 0.5 12 17.5

---------+---------------------------------

12 | 2.8e+10

| 1.000

|

17.5 | 1.5e+11 1.2e+11

| 0.376 0.697

|

19 | 2.8e+10 -1.7e+08 -1.2e+11

| 1.000 1.000 0.694

Section 2.9 - viabmm0.5xcmm hours, bonferroni means st

| Summary of mm0.5 x cmm12

| x cmm17.5 x cmm19

hours | Mean Std. Dev.

------------+------------------------

0.5 | 3.617e+08 27537853

12 | 4.760e+10 4.980e+09

17.5 | 5.347e+10 1.537e+10

19 | 2.840e+10 1.622e+09

------------+------------------------

Total | 3.246e+10 2.272e+10

Analysis of Variance

Source SS df MS F Prob > F

------------------------------------------------------------------------

Between groups 5.1519e+21 3 1.7173e+21 26.04 0.0002

Within groups 5.2755e+20 8 6.5944e+19

------------------------------------------------------------------------

Total 5.6794e+21 11 5.1631e+20

Bartlett's test for equal variances: chi2(3) = 23.6617 Prob>chi2 = 0.000

Section 2.10 - Comparison of mm0.5 x cmm12 x cmm17.5 x cmm19 by hours

(Bonferroni)

Row Mean-|

Col Mean | 0.5 12 17.5

---------+---------------------------------

12 | 4.7e+10

| 0.001

|

17.5 | 5.3e+10 5.9e+09

| 0.000 1.000

|

19 | 2.8e+10 -1.9e+10 -2.5e+10

| 0.017 0.120 0.032

viabmm12xcmm hours, bonferroni means st

| Summary of cmm0.5 x mm12

| x cmm17.5 x cmm19

hours | Mean Std. Dev.

------------+------------------------

0.5 | 4.250e+08 30413813

12 | 4.615e+10 6.178e+09

17.5 | 5.347e+10 1.537e+10

19 | 2.840e+10 1.622e+09

------------+------------------------

Total | 3.211e+10 2.250e+10

Analysis of Variance

Source SS df MS F Prob > F

------------------------------------------------------------------------

Between groups 5.0128e+21 3 1.6709e+21 24.12 0.0002

Within groups 5.5429e+20 8 6.9286e+19

------------------------------------------------------------------------

Total 5.5671e+21 11 5.0610e+20

Bartlett's test for equal variances: chi2(3) = 22.9465 Prob>chi2 = 0.000

Section 2.11 - Comparison of cmm0.5 x mm12 x cmm17.5 x cmm19 by hours

(Bonferroni)

Row Mean-|

Col Mean | 0.5 12 17.5

---------+---------------------------------

12 | 4.6e+10

| 0.001

|

17.5 | 5.3e+10 7.3e+09

| 0.000 1.000

|

19 | 2.8e+10 -1.8e+10 -2.5e+10

| 0.020 0.186 0.037

Section 2.12 - viabmm17.5xcmm hours, bonferroni means st

| Summary of cmm0.5 x

| cmm12 x mm17.5 x cmm19

hours | Mean Std. Dev.

------------+------------------------

0.5 | 4.250e+08 30413813

12 | 4.760e+10 4.980e+09

17.5 | 4.767e+10 3.924e+09

19 | 2.840e+10 1.622e+09

------------+------------------------

Total | 3.102e+10 2.038e+10

Analysis of Variance

Source SS df MS F Prob > F

------------------------------------------------------------------------

Between groups 4.4848e+21 3 1.4949e+21 139.63 0.0000

Within groups 8.5654e+19 8 1.0707e+19

------------------------------------------------------------------------

Total 4.5704e+21 11 4.1549e+20

Bartlett's test for equal variances: chi2(3) = 15.8175 Prob>chi2 = 0.001

Section 2.13 - Comparison of cmm0.5 x cmm12 x mm17.5 x cmm19 by hours

(Bonferroni)

Row Mean-|

Col Mean | 0.5 12 17.5

---------+---------------------------------

12 | 4.7e+10

| 0.000

|

17.5 | 4.7e+10 6.7e+07

| 0.000 1.000

|

19 | 2.8e+10 -1.9e+10 -1.9e+10

| 0.000 0.001 0.001

viabmm19xcmm hours, bonferroni means st

| Summary of cmm0.5 x

| cmm12 x cmm17.5 x mm19

hours | Mean Std. Dev.

------------+------------------------

0.5 | 4.250e+08 30413813

12 | 4.760e+10 4.980e+09

17.5 | 5.347e+10 1.537e+10

19 | 2.798e+10 7.286e+08

------------+------------------------

Total | 3.237e+10 2.271e+10

Analysis of Variance

Source SS df MS F Prob > F

------------------------------------------------------------------------

Between groups 5.1502e+21 3 1.7167e+21 26.24 0.0002

Within groups 5.2335e+20 8 6.5418e+19

------------------------------------------------------------------------

Total 5.6736e+21 11 5.1578e+20

Bartlett's test for equal variances: chi2(3) = 25.9303 Prob>chi2 = 0.000

Section 2.14 - Comparison of cmm0.5 x cmm12 x cmm17.5 x mm19 by hours

(Bonferroni)

Row Mean-|

Col Mean | 0.5 12 17.5

---------+---------------------------------

12 | 4.7e+10

| 0.001

|

17.5 | 5.3e+10 5.9e+09

| 0.000 1.000

|

19 | 2.8e+10 -2.0e+10 -2.5e+10

| 0.019 0.107 0.029

Chapter 3 - GST

**Descriptive Statistics for Each Value of Crosstab Variable**

|  | Obs | Total | Mean | Variance | Std Dev |
| --- | --- | --- | --- | --- | --- |
| 12 | 6 | ,0462 | ,0077 | ,0000 | ,0013 |
| 17 | 6 | ,1074 | ,0179 | ,0007 | ,0257 |
| 19 | 6 | ,0842 | ,0140 | ,0000 | ,0025 |

|  | Minimum | 25% | Median | 75% | Maximum | Mode |
| --- | --- | --- | --- | --- | --- | --- |
| 12 | 0,0069 | 0,0071 | 0,0073 | 0,0073 | 0,0104 | 0,0069 |
| 17 | 0,0071 | 0,0071 | 0,0074 | 0,0081 | 0,0704 | 0,0071 |
| 19 | 0,0099 | 0,0130 | 0,0142 | 0,0162 | 0,0167 | 0,0099 |

**ANOVA, a Parametric Test for Inequality of Population Means**

(For normally distributed data only)

| Variation | SS | df | MS | F statistic |
| --- | --- | --- | --- | --- |
| Between | 0,0003 | 2 | 0,0002 | 0,7146 |
| Within | 0,0033 | 15 | 0,0002 |  |
| Total | 0,0037 | 17 |  |  |

**P-value = 0,5053 (= do STATA)**

**Bartlett’s Test for Inequality of Population Variances**

| Bartlett’s chi square= | 33,9312 | df=2 | **P value=0,0000** |
| --- | --- | --- | --- |

A small p-value (e.g., less than 0.05 suggests that the variances **are not homogeneous and that the ANOVA may not be appropriate.**

**Mann-Whitney/Wilcoxon Two-Sample Test (Kruskal-Wallis test for two groups)**

| Kruskal-Wallis H (quivalente to Chi square) = | 8,0351 |
| --- | --- |
| Degrees of freedom = | 2 |
| **P value =** | **0,0180** |

Chapter 4 - MDA

Section 4.1 - mda12 media12 hours, bonferroni means st

1=mm x |

2=mmm x | Summary of 3 mda 12

3=cmm | Mean Std. Dev.

------------+------------------------

1 | 1.20125 .14840117

2 | 1.1625 .08948928

3 | 1.1366667 .17897858

------------+------------------------

Total | 1.1695455 .12713092

Analysis of Variance

Source SS df MS F Prob > F

------------------------------------------------------------------------

Between groups .007462312 2 .003731156 0.19 0.8277

Within groups .154160384 8 .019270048

------------------------------------------------------------------------

Total .161622696 10 .01616227

Bartlett's test for equal variances: chi2(2) = 1.0371 Prob>chi2 = 0.595

Comparison of 3 mda hours 12 by 1=mm x 2=mmm x 3=cmm

(Bonferroni)

Row Mean-|

Col Mean | 1 2

---------+----------------------

2 | -.03875

| 1.000

|

3 | -.064583 -.025833

| 1.000 1.000

Section 4.2 - mda17.5 media17.5 hours, bonferroni means st

1=mm x |

2=mmm x | Summary of 3 mda17.5

3=cmm | Mean Std. Dev.

------------+------------------------

1 | 1.6275 .26846789

2 | .98166668 .08948931

3 | .82666665 .08948931

------------+------------------------

Total | 1.1935 .41365613

Analysis of Variance

Source SS df MS F Prob > F

------------------------------------------------------------------------

Between groups 1.29174417 2 .645872083 18.21 0.0017

Within groups .248258377 7 .035465482

------------------------------------------------------------------------

Total 1.54000254 9 .171111394

Bartlett's test for equal variances: chi2(2) = 3.1916 Prob>chi2 = 0.203

Comparison of 3 mda17.5 by 1=mm x 2=mmm x 3=cmm

(Bonferroni)

Row Mean-|

Col Mean | 1 2

---------+----------------------

2 | -.645833

| 0.008

|

3 | -.800833 -.155

| 0.003 1.000

Section 4.3 - mda19 media19 hours, bonferroni means st

1=mm x |

2=mmm x | Summary of 3 mda19

3=cmm | Mean Std. Dev.

------------+------------------------

1 | .93000001 .10960157

2 | .69749999 .08948928

3 | 1.271 .06931809

------------+------------------------

Total | .98535714 .25510529

Analysis of Variance

Source SS df MS F Prob > F

------------------------------------------------------------------------

Between groups .754728243 2 .377364122 45.47 0.0000

Within groups .091295003 11 .008299546

------------------------------------------------------------------------

Total .846023246 13 .065078711

Bartlett's test for equal variances: chi2(2) = 0.7247 Prob>chi2 = 0.696

Comparison of 3 mda19 by 1=mm x 2=mmm x 3=cmm

(Bonferroni)

Row Mean-|

Col Mean | 1 2

---------+----------------------

2 | -.2325

| 0.009

|

3 | .341 .5735

| 0.000 0.000

Section 4.4 - mdammxmm time_0, bonferroni means st

12mm x |

17.5mm x | Summary of media

19mm | Mean Std. Dev.

------------+------------------------

12 | 1.20125 .14840117

17.5 | 1.6275 .26846789

19 | .93000001 .10960157

------------+------------------------

Total | 1.2280769 .34346602

Analysis of Variance

Source SS df MS F Prob > F

------------------------------------------------------------------------

Between groups 1.08528314 2 .54264157 16.43 0.0007

Within groups .330343776 10 .033034378

------------------------------------------------------------------------

Total 1.41562692 12 .11796891

Bartlett's test for equal variances: chi2(2) = 2.5722 Prob>chi2 = 0.276

Comparison of media by 12mm x 17.5mm x 19mm

(Bonferroni)

Row Mean-|

Col Mean | 12 17.5

---------+----------------------

17.5 | .42625

| 0.023

|

19 | -.27125 -.6975

| 0.151 0.001

Section 4.5 - mm12xmm19 tmpmm12x19, bonferroni means st

| Summary of media

mm12 x mm19 | Mean Std. Dev.

------------+------------------------

12 | 1.20125 .14840117

19 | .93000001 .10960157

------------+------------------------

Total | 1.0505556 .18628682

Analysis of Variance

Source SS df MS F Prob > F

------------------------------------------------------------------------

Between groups .163503484 1 .163503484 10.03 0.0158

Within groups .114118745 7 .016302678

------------------------------------------------------------------------

Total .277622229 8 .034702779

Bartlett's test for equal variances: chi2(1) = 0.2784 Prob>chi2 = 0.598

Comparison of media by mm12 x mm19

(Bonferroni)

Row Mean-|

Col Mean | 12

---------+-----------

19 | -.27125

| 0.016

Section 4.6 - mdammxmmm time_1, bonferroni means st

12mm x |

17.5mmm x | Summary of mdammxmmm

19mmm | Mean Std. Dev.

------------+------------------------

12 | 1.20125 .14840117

17.5 | .98166668 .08948931

19 | .69749999 .08948928

------------+------------------------

Total | .95818183 .24817608

Analysis of Variance

Source SS df MS F Prob > F

------------------------------------------------------------------------

Between groups .509803275 2 .254901637 19.22 0.0009

Within groups .10611039 8 .013263799

------------------------------------------------------------------------

Total .615913664 10 .061591366

Bartlett's test for equal variances: chi2(2) = 0.8535 Prob>chi2 = 0.653

Comparison of mdammxmmm by 12mm x 17.5mmm x 19mmm

(Bonferroni)

Row Mean-|

Col Mean | 12 17.5

---------+----------------------

17.5 | -.219583

| 0.111

|

19 | -.50375 -.284167

| 0.001 0.036

Section 4.7 - mm12x17.5mmm tpmm12x17.5 3m, bonferroni means st

12mm x | Summary of media

17.5mmm | Mean Std. Dev.

------------+------------------------

12 | 1.20125 .14840117

17.5 | .98166668 .08948931

------------+------------------------

Total | 1.1071429 .16570196

Analysis of Variance

Source SS df MS F Prob > F

------------------------------------------------------------------------

Between groups .08265744 1 .08265744 5.03 0.0749

Within groups .082085399 5 .01641708

------------------------------------------------------------------------

Total .164742839 6 .02745714

Bartlett's test for equal variances: chi2(1) = 0.4578 Prob>chi2 = 0.499

Comparison of media by 12mm x 17.5mmm

(Bonferroni)

Row Mean-|

Col Mean | 12

---------+-----------

17.5 | -.219583

| 0.075

Section 4.8 - mdammxcmm time_2, bonferroni means st

12mm x |

17.5cmm x | Summary of mdammxcmm

19cmm | Mean Std. Dev.

------------+------------------------

12 | 1.20125 .14840117

17.5 | .82666665 .08948931

19 | 1.271 .06931809

------------+------------------------

Total | 1.1366667 .21245677

Analysis of Variance

Source SS df MS F Prob > F

------------------------------------------------------------------------

Between groups .395211289 2 .197605644 17.56 0.0008

Within groups .101305392 9 .011256155

------------------------------------------------------------------------

Total .49651668 11 .04513788

Bartlett's test for equal variances: chi2(2) = 1.7834 Prob>chi2 = 0.410

Comparison of mdammxcmm by 12mm x 17.5cmm x 19cmm

(Bonferroni)

Row Mean-|

Col Mean | 12 17.5

---------+----------------------

17.5 | -.374583

| 0.004

|

19 | .06975 .444333

| 1.000 0.001

Section 4.9 - mdammm tmpmmm, bonferroni means st

12mmm x |

17.5mmm x | Summary of media

19mmm | Mean Std. Dev.

------------+------------------------

12 | 1.1625 .08948928

17.5 | .98166668 .08948931

19 | .69749999 .08948928

------------+------------------------

Total | .94409092 .22412964

Analysis of Variance

Source SS df MS F Prob > F

------------------------------------------------------------------------

Between groups .438274307 2 .219137153 27.36 0.0003

Within groups .064066655 8 .008008332

------------------------------------------------------------------------

Total .502340962 10 .050234096

Bartlett's test for equal variances: chi2(2) = 0.0000 Prob>chi2 = 1.000

Comparison of media by 12mmm x 17.5mmm x 19mmm

(Bonferroni)

Row Mean-|

Col Mean | 12 17.5

---------+----------------------

17.5 | -.180833

| 0.088

|

19 | -.465 -.284167

| 0.000 0.010

Section 4.10 - mda12x17.5 mmm12x17.5, bonferroni means st

mmm12 x | Summary of media

mmm17.5 | Mean Std. Dev.

------------+------------------------

12 | 1.1625 .08948928

17.5 | .98166668 .08948931

------------+------------------------

Total | 1.085 .12655697

Analysis of Variance

Source SS df MS F Prob > F

------------------------------------------------------------------------

Between groups .056058337 1 .056058337 7.00 0.0457

Within groups .040041664 5 .008008333

------------------------------------------------------------------------

Total .096100001 6 .016016667

Bartlett's test for equal variances: chi2(1) = 0.0000 Prob>chi2 = 1.000

Comparison of media by mmm12 x mmm17.5

(Bonferroni)

Row Mean-|

Col Mean | 12

---------+-----------

17.5 | -.180833

| 0.046

Section 4.11 - mdacmm tmpmmc, bonferroni means st

12mmc x |

17.5mmc x | Summary of media

19mmc | Mean Std. Dev.

------------+------------------------

12 | 1.1366667 .17897858

17.5 | .82666665 .08948931

19 | 1.271 .06931809

------------+------------------------

Total | 1.1131818 .21720121

Analysis of Variance

Source SS df MS F Prob > F

------------------------------------------------------------------------

Between groups .372460334 2 .186230167 15.00 0.0020

Within groups .099303334 8 .012412917

------------------------------------------------------------------------

Total .471763667 10 .047176367

Bartlett's test for equal variances: chi2(2) = 2.3383 Prob>chi2 = 0.311

Comparison of media by 12mmc x 17.5mmc x 19mmc

(Bonferroni)

Row Mean-|

Col Mean | 12 17.5

---------+----------------------

17.5 | -.31

| 0.028

|

19 | .134333 .444333

| 0.412 0.002

Section 4.12 - mda12x19 mmc12x19, bonferroni means st

12mmc x | Summary of media

19mmc | Mean Std. Dev.

------------+------------------------

12 | 1.1366667 .17897858

19 | 1.271 .06931809

------------+------------------------

Total | 1.220625 .12935105

Analysis of Variance

Source SS df MS F Prob > F

------------------------------------------------------------------------

Between groups .033835206 1 .033835206 2.44 0.1695

Within groups .083286661 6 .01388111

------------------------------------------------------------------------

Total .117121866 7 .016731695

Bartlett's test for equal variances: chi2(1) = 2.1525 Prob>chi2 = 0.142

Comparison of media by 12mmc x 19mmc

(Bonferroni)

Row Mean-|

Col Mean | 12

---------+-----------

19 | .134333

| 0.169

Section 4.13 - mmm12xmmc tmp3m12xmmc, bonferroni means st

mmm12 x |

17.5mmc x | Summary of media

19mmc | Mean Std. Dev.

------------+------------------------

12 | 1.1625 .08948928

17.5 | .82666665 .08948931

19 | 1.271 .06931809

------------+------------------------

Total | 1.12375 .19964885

Analysis of Variance

Source SS df MS F Prob > F

------------------------------------------------------------------------

Between groups .37919462 2 .18959731 28.79 0.0001

Within groups .059261657 9 .006584629

------------------------------------------------------------------------

Total .438456278 11 .039859662

Bartlett's test for equal variances: chi2(2) = 0.2423 Prob>chi2 = 0.886

Comparison of media by mmm12 x 17.5mmc x 19mmc

(Bonferroni)

Row Mean-|

Col Mean | 12 17.5

---------+----------------------

17.5 | -.335833

| 0.001

|

19 | .1085 .444333

| 0.232 0.000

Section 4.14 - mmm12xmmc19 tmp12x19, bonferroni means st

12mmm x | Summary of media

19mmc | Mean Std. Dev.

------------+------------------------

12 | 1.1625 .08948928

19 | 1.271 .06931809

------------+------------------------

Total | 1.2227778 .09314339

Analysis of Variance

Source SS df MS F Prob > F

------------------------------------------------------------------------

Between groups .026160546 1 .026160546 4.23 0.0786

Within groups .043244984 7 .006177855

------------------------------------------------------------------------

Total .06940553 8 .008675691

Bartlett's test for equal variances: chi2(1) = 0.1977 Prob>chi2 = 0.657

Comparison of media by 12mmm x 19mmc

(Bonferroni)

Row Mean-|

Col Mean | 12

---------+-----------

19 | .1085

| 0.079

Section 4.15 - mmc12xmmm tmmc12xmmm, bonferroni means st

mmc12 x |

mmm17.5 x | Summary of media

mmm19 | Mean Std. Dev.

------------+------------------------

12 | 1.1366667 .17897858

17.5 | .98166668 .08948931

19 | .69749999 .08948928

------------+------------------------

Total | .9145 .22461635

Analysis of Variance

Source SS df MS F Prob > F

------------------------------------------------------------------------

Between groups .349964202 2 .174982101 11.77 0.0058

Within groups .104108332 7 .014872619

------------------------------------------------------------------------

Total .454072534 9 .050452504

Bartlett's test for equal variances: chi2(2) = 1.3023 Prob>chi2 = 0.521

Comparison of media by mmc12 x mmm17.5 x mmm19

(Bonferroni)

Row Mean-|

Col Mean | 12 17.5

---------+----------------------

17.5 | -.155

| 0.491

|

19 | -.439167 -.284167

| 0.007 0.056

Section 4.16 - mmm17.5x3m19 t3m17.5x3m19, bonferroni means st

mmm17.5 x | Summary of media

19mmm | Mean Std. Dev.

------------+------------------------

17.5 | .98166668 .08948931

19 | .69749999 .08948928

------------+------------------------

Total | .81928572 .17246809

Analysis of Variance

Source SS df MS F Prob > F

------------------------------------------------------------------------

Between groups .138429788 1 .138429788 17.29 0.0088

Within groups .040041664 5 .008008333

------------------------------------------------------------------------

Total .178471452 6 .029745242

Bartlett's test for equal variances: chi2(1) = 0.0000 Prob>chi2 = 1.000

Comparison of media by mmm17.5 x 19mmm

(Bonferroni)

Row Mean-|

Col Mean | 17.5

---------+-----------

19 | -.284167

| 0.009
